# SensitiveCancerGPT: Leveraging Generative Large Language Model on Structured Omics Data to Optimize Drug Sensitivity Prediction

**DOI:** 10.1101/2025.02.27.640661

**Published:** 2025-03-03

**Authors:** Shaika Chowdhury, Sivaraman Rajaganapathy, Lichao Sun, Liewei Wang, Ping Yang, James R Cerhan, Nansu Zong

**Affiliations:** 1 Department of Artificial Intelligence and Informatics Research, Mayo Clinic, Rochester, MN;; 2 Lehigh University, Bethlehem, PA;; 3 Department of Quantitative Health Sciences, Mayo Clinic, Rochester, MN;; 4 Department of Molecular Pharmacology and Experimental Therapeutics, Mayo Clinic, Rochester, MN.

## Abstract

**Objective:**

The fast accumulation of vast pharmacogenomics data of cancer cell lines provide unprecedented opportunities for drug sensitivity prediction (DSP), a crucial prerequisite for the advancement of precision oncology. Recently, Generative Large Language Models (LLM) have demonstrated performance and generalization prowess across diverse tasks in the field of natural language processing (NLP). However, the structured format of the pharmacogenomics data poses challenge for the utility of LLM in DSP. Therefore, the objective of this study is multi-fold: to adapt prompt engineering for structured pharmacogenomics data toward optimizing LLM’s DSP performance, to evaluate LLM’s generalization in real-world DSP scenarios, and to compare LLM’s DSP performance against that of state-of-the-science baselines.

**Methods:**

We systematically investigated the capability of the Generative Pre-trained Transformer (GPT) as a DSP model on four publicly available benchmark pharmacogenomics datasets, which are stratified by five cancer tissue types of cell lines and encompass both oncology and non-oncology drugs. Essentially, the predictive landscape of GPT is assessed for effectiveness on the DSP task via four learning paradigms: zero-shot learning, few-shot learning, fine-tuning and clustering pretrained embeddings. To facilitate GPT in seamlessly processing the structured pharmacogenomics data, domain-specific novel prompt engineering is employed by implementing three prompt templates (i.e., Instruction, Instruction-Prefix, Cloze) and integrating pharmacogenomics-related features into the prompt. We validated GPT’s performance in diverse real-world DSP scenarios: cross-tissue generalization, blind tests, and analyses of drug-pathway associations and top sensitive/resistant cell lines. Furthermore, we conducted a comparative evaluation of GPT against multiple Transformer-based pretrained models and existing DSP baselines.

**Results:**

Extensive experiments on the pharmacogenomics datasets across the five tissue cohorts demonstrate that fine-tuning GPT yields the best DSP performance (28% F1 increase, p-value= 0.0003) followed by clustering pretrained GPT embeddings (26% F1 increase, p-value= 0.0005), outperforming GPT in-context learning (i.e., few-shot). However, GPT in the zero-shot setting had a big F1 gap, resulting in the worst performance. Within the scope of prompt engineering, performance enhancement was achieved by directly instructing GPT about the DSP task and resorting to a concise context format (i.e., instruction-prefix), leading to F1 performance gain of 22% (p-value=0.02); while incorporation of drug-cell line prompt context derived from genomics and/or molecular features further boosted F1 score by 2%. Compared to state-of-the-science DSP baselines, GPT significantly asserted superior mean F1 performance (16% gain, p-value<0.05) on the GDSC dataset. In the cross-tissue analysis, GPT showcased comparable generalizability to the within-tissue performances on the GDSC and PRISM datasets, while statistically significant F1 performance improvements on the CCLE (8%, p-value=0.001) and DrugComb (19%, p-value=0.009) datasets. Evaluation on the challenging blind tests suggests GPT’s competitiveness on the CCLE and DrugComb datasets compared to random splitting. Furthermore, analyses of the drug-pathway associations and log probabilities provided valuable insights that align with previous DSP findings.

**Conclusion:**

The diverse experiment setups and in-depth analysis underscore the importance of generative LLM, such as GPT, as a viable in silico approach to guide precision oncology.

**Availability:**

https://github.com/bioIKEA/SensitiveCancerGPT

## 1. Introduction

Cancer is a complex genetic disease that originates from the accumulation of gene mutations within a cell and is ranked as the second leading cause of death in the United States, according to the American Cancer Society (Siegel et al. 2023). Given the tumor heterogeneity arising from the genetic variations among patients even with the same cancer type (Bedard et al. 2013), substantial differences in the anti-cancer drug response can be expected, thereby highlighting the urgent need for targeted therapies. Owing to the high cost and time associated with developing and validating anti-cancer drugs in clinical trials - which is further exacerbated by the 96% failure rate (Kola et al. 2004) – it is imperative to develop preclinical computational models that can accurately predict whether a cell line is sensitive or resistant to a particular drug. The availability of genomic profiles of cancer cell lines (Barretina et al. 2012, Yang et al. 2012) collected via high-throughput screening technologies offers feasible resources to develop robust drug response models and identify the important biomarkers predictive of drug sensitivity.

The advent of cutting-edge machine learning methods, especially deep learning-based ones, coupled with large-scale pharmacogenomics datasets, has ushered in the development of data-driven computational methods for the effective prediction of cancer drug response (Li et al. 2019, Chiu et al. 2019, Zuo et al. 2021, Manica et al. 2019 and G et al. 2020) (Literature Review presented in Supplementary Note 1). These methods formalize DSP either into a regression or classification problem contingent on the prediction of a continuous (i.e., IC50) or categorical (i.e., sensitive or resistant) output, respectively. Modeling this problem involves feature extraction from the drug’s molecular properties and cell line’s genomic characterizations to learn meaningful representations. Although deep learning can model high-dimensional biological data to capture the complex, non-linear patterns (Baptista et al. 2021, Stephenson et al. 2019), it is not well suited for omics data as deep learning has shown to fare poorly with sub-par performance on structured tabular data (Borisov et al. 2022, Shwartz-Ziv et al. 2022).

Recently, generative large language models (LLM) have exhibited unprecedented capabilities on a broad array of NLP tasks (Chang et al. 2023, Zhang et al. 2023, Liu et al. 2023). LLMs are first pretrained on large text corpora to acquire knowledge of the general language features and then fine-tuned on domain-specific datasets for downstream tasks, known as the *pretrain-and-finetune* paradigm. More recently, *pretrain, prompt and predict* has emerged as a new paradigm for the GPT (Generative Pre-trained Transformer) family of the autoregressive LLM model, which can be applied for downstream tasks using task-specific input templates, known as prompts, under the zero or few shot settings without updating the model parameters. Unlike standard deep learning models which focus on automatic feature engineering from the data for optimization on downstream tasks, GPT-based LLM models exploit input engineering that leverages textual prompts to reformulate downstream tasks through the incorporation of task-specific details.

Recent studies have noted the potential of GPT in the biomedical domain focused on information extraction (IE) and NLP tasks that entail processing unstructured biomedical texts (Gutierrez et al. 2022, Moradi et al. 2021). The applicability of GPT for DSP, however, is challenged by the structured pharmacogenomics databases, which are organized in a two-dimensional grid format as tables of rows and columns. Given the inherent heterogeneity of this tabular data with mixed column data types, adapting GPT for DSP necessitates specialized prompt design. This is a non-trivial undertaking as it involves linearizing the structured feature attributes, which could be accompanied by high-dimensional genomic values, into a natural language sentence that would elicit the most accurate drug response.

**Figure 1.**
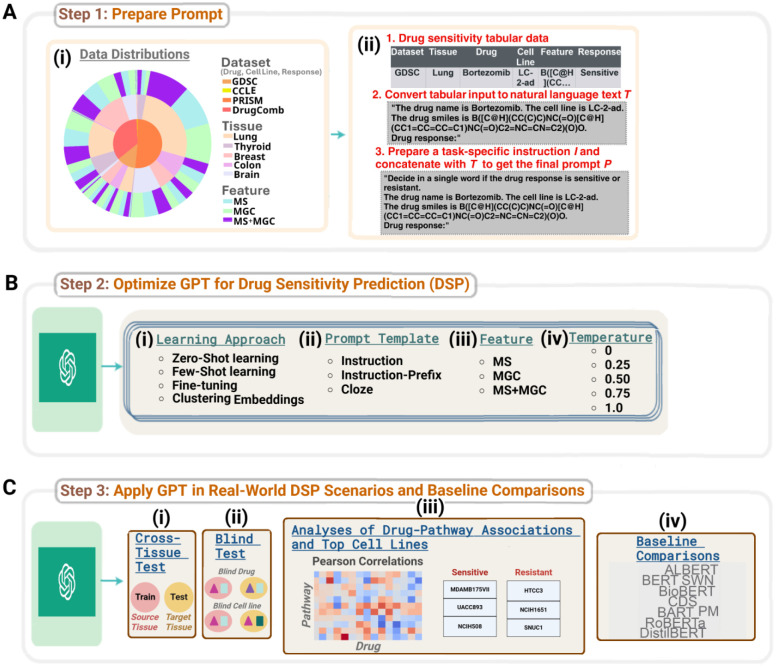
An overview of our proposed SensitiveCancerGPT framework. (A) (i) The statistics of the pharmacogenomics datasets presented as a nested pie plot. The total distribution of the cell line drug response within each dataset is shown as the innermost plot, the tissue distributions within each dataset as the middle plot and the feature distributions within each tissue cohort as the outermost plot. (ii) Workflow of prompt preparation from structured pharmacogenomics data. Using the column names and corresponding values in the tabular data, we first convert each row to a natural language text *T*. Note that the last column is left blank for the model to predict. We then prepare a task-specific instruction *I* and concatenate it with *T* to get the final prompt *P*. (B) To optimize GPT’s performance for the drug sensitivity prediction task (DSP), we evaluate on four different methodological factors: (i) learning approach, (ii) prompt template, (iii) feature and (iv) temperature. (C) We assess GPT’s generalization capability for DSP under diverse real-world experimental settings: (i) cross-tissue evaluation, (ii) blind tests, (iii) analyses of drug-pathway associations and top cell lines and (iv) baseline comparisons.

This study aims to bridge the current research gap in the application of GPT for predicting cancer drug response based on structured pharmacogenomics data. As illustrated in Figure 1, we utilize prompt engineering to design relevant templates that demonstrate how tabular features of drug and cell line can be integrated into GPT’s input prompt, facilitating GPT’s performance optimization for DSP through evaluation under a wide range of learning approaches. We further investigate GPT’s adaptability in various experimental settings to realize its full potential for DSP. To the best of our knowledge, this work marks the first comprehensive study of GPT’s deployment on structured pharmacogenomics data for the DSP task. The promising performance of GPT offers a new direction in advancing AI-based precision oncology through the intersection of generative LLM with structured pharmacogenomics data.

### 1.1. Statement of Significance

#### Problem:

Drug sensitivity prediction (DSP) aims to predict drug response in cancer patients for personalized treatment. Large-scale pharmacogenomics datasets accumulated in high-throughput screening technologies provide a valuable resource to fulfill this task. As a result, great computational efforts have been made to analyze these data by using machine learning to build predictive models for DSP that would benefit anti-cancer therapeutics.

#### What is already known:

Generative large language models (LLM) have exhibited exceptional ability in comprehending and generating unstructured textual data in the general domain, while they have been hardly applied on structured pharmacogenomics data to facilitate drug response prediction.

#### What this paper adds:

This study investigates the diverse learning capabilities of the Generative Pre-trained Transformer (GPT) to analyze the genomic and molecular patterns of drug-cell lines in structured pharmacogenomics databases by introducing domain-specific novel prompt engineering to ensure effective DSP performance. We demonstrate the feasibility of GPT through comprehensive evaluations under different experimental settings including (1) cross-tissue generalization, (2) blind tests, (3) baseline comparisons and (4) analyses of drug-pathway associations and top sensitive/resistant cell lines.

## 2. Materials and methods

### 2.1. Benchmark pharmacogenomics datasets

We conduct our experiments on four publicly available pharmacogenomics resources – GDSC, CCLE, DrugComb and PRISM – which we categorize according to the respective indication of the drugs into principally two different types, oncology and non-oncology. GDSC, CCLE and DrugComb were screened against oncology drugs to test the anti-cancer sensitivity of cell lines, while PRISM screened predominantly non-oncology drugs for anti-cancer repurposing. The Genomics of Drugs Sensitivity in Cancer (GDSC) (Yang et al. 2012) comprises screening response data from two phases: GDSC1 includes 987 cancer cell lines and 320 compounds; GDSC2 includes an additional 809 cancer cell lines and 175 compounds. We use GDSC2, in which cancer cell lines are characterized by genetic features, such as the mutation state. The Cancer cell line Encyclopedia (CCLE) (Barretina et al. 2012) contains a large-scale genomic data (e.g., gene expression) for 947 human cancer cell lines and response data for around 500 of the cell lines to 24 drug compounds. The DrugComb (Zagidullin et al. 2019, Zheng et al. 2021) dataset includes data on synergy and sensitivity of drug combinations. We use the single drug sensitivity data that contains 717,684 single drug screenings from 37 studies. For each drug-drug sample in the dataset, we pair each drug with the cell line to form two separate drug-cell line inputs and label each with the corresponding single drug IC50 score. The secondary PRISM Repurposing dataset (Corsello et al. 2020) is a drug repurposing database that includes 1448 drug compounds, out of which majority were for non-oncology purposes, screened against 499 cell lines. Detailed descriptions of the datasets can be found in Supplementary Note 2.

#### 2.1.1. Stratification by cancer type

We stratify the drug-cell line pairs within each dataset by the tissue-of-origin to reinforce GPT’s DSP capability across a diverse spectrum of patients with different cancer types. In particular, we consider the following five tissue types which were available across all four datasets: lung, thyroid, breast, brain, colon/stomach. The data distributions are shown in Figure 1A(i) (Supplementary Table 1) and further details are provided in Supplementary Note 3.

### 2.2. Generative LLM used

Large Language Models (LLM) refer to scaled-up Transformer-based pretrained language models. The Generative Pre-trained Transformer (GPT) variants from OpenAI are decoder-based autoregressive family of LLMs and includes, at the time of this study, GPT-3, GPT-3.5 and GPT-4. These models are accessible through OpenAI API at a usage fees. Although the successor LLMs of GPT-3 are known to demonstrate stronger capabilities, they incur higher inference cost. To balance the cost-effectiveness and DSP performance of GPT, we carried out an empirical analysis (Supplementary Note 4) comparing evaluations between GPT-3, GPT-3.5 and GPT-4. We found that GPT-3.5 and GPT-4 performed the same as GPT-3 with no additional improvements. Hence, we opt for GPT-3 in all our experiments.

### 2.3. Learning approach

We employ four different paradigms to adapt GPT-3 for downstream DSP analysis: (i) Fine-tuning trains and then tests the pretrained GPT-3 model on prompt-completion pairs, (ii) zero-shot learning directly runs the pretrained GPT-3 model on textual prompt inputs from the test set to generate the drug sensitivity response during inference, (iii) few-shot is akin to zero shot but additionally integrates few examples into the prompt and (iv) clustering embeddings refines the GPT-3-derived textual embeddings by fitting a Bayesian Gaussian Mixture Model (BGMM) (Bishop C 2006). Please refer to Supplementary Note 5 for detailed descriptions.

### 2.4. Prompt engineering

Prompting GPT-3 for DSP refers to providing the relevant pharmacogenomics knowledge via carefully designed textual templates to guide the model’s response generation. The prompt preparation steps are illustrated in Figure 1A(ii). The pharmacogenomics data is formatted as a table of M rows of drug-cell line instances and N columns. Note that the first row defines the headers corresponding to the column names. We employ relevant prompt engineering of the tabular pharmacogenomics data specific to each learning approach.

#### 2.4.1. Zero-shot

A standard prompt *P* could be crafted with the following elements: task-specific instruction *I*, input *T*, context *C* and output placeholder *R*. We perform table serialization (Hegselmann et al. 2023) by converting each row in the test set to a natural language text *T* based on the headers and the corresponding column values. Note that the last column’s value is considered as *R* and left blank. We then prepare *I* and concatenate it with *T* to get the final prompt *P*, which is directly inputted into GPT-3 to generate the drug response.

#### 2.4.2. Few-shot

We expand the zero-shot prompt template *P* with context *C*. The context *C* constitutes *k* pairs of drug-cell line and ground truth response examples. We experiment with three different prompt design templates. We devise the three templates based on the following: inclusion of *I*, format of *T* and *C*, and order of *R*. As such, *instruction prompt* includes *I*, has a sentence format for *T* and *C*, and places *R* at the end: *P = I + T + C + R*. Here, the + sign denotes concatenation followed by a new line. The *instruction*-p*refix prompt* is similar to instruction prompt, however, *T* and *C* have a concise format as we prepend the headers to the respective column values with colon prefix: *P = I + T’ + C’ + R*, where *‘* indicates the concise format. Lastly, *cloze prompt* does not include *I*, inserts *R* within *T* (analogous to filling in the blank), and maintains sentence format for *T* and *C*: *P = TR + C*. We illustrate each prompt template with an example in Supplementary Figure 2.

#### 2.4.3. Fine-tuning

We prepare the training and test data in the form of prompt-completion pairs. The prompt is represented using a concise prompt template similar to instruction-prefix and the completion is the ground truth sensitive/resistant label.

### 2.5. Prompt features

The content within *T* and *C* could be grounded with pharmacogenomics knowledge derived from the ‘Feature’ column. We assess the informativeness of two types of features - *molecular structure information* (*MS*) and *additional molecular or genomic context* (*MGC*) - that are integrated into the prompt template with the drug-cell line input (*Basic Information* (*BI*)). This leads to three feature groups in total (BI + MS, BI + MGC, BI + MS + MGC), that are evaluated per dataset. The feature descriptions can be found in Supplementary Note 6 and the feature-ablated dataset sizes are shown in Supplementary Table 1.

## 3. Experiment Setup

### 3.1 Task Formulation

We formulate drug sensitivity prediction as a binary classification problem to predict if a drug-cell line pair is *sensitive* or *resistant*. For the drug-cell line pairs in each dataset, we adopt the corresponding IC50 (the half maximal inhibitory concentration) values to gauge the drug sensitivity. To convert the IC50 drug response values to binary labels, we adopt the strategy employed in (Chang et al. 2018) and set a fixed threshold θ = −2, where ln(IC50) < θ is considered sensitive, and resistant otherwise.

### 3.2. Evaluations

#### 3.2.1. Optimization of GPT’s DSP performance

We investigate the influence of four methodological factors that shape the DSP performance. These include learning approach, prompt template, feature and temperature. For learning approach, prompt template and feature, we use the settings outlined in **Sections 2.3**, **2.4.2** and **2.5**, respectively. For GPT-3’s temperature setting, we evaluate on values in increment of 0.25 between 0 and 1. Analysis is carried out separately on each factor by evaluating GPT-3 relative to the settings associated with that factor, while the other three factors’ settings are kept fixed: fine-tuning for learning approach, instruction-prefix for prompt template, MS for feature, and 0 for temperature. Only in the assessment of prompt template, we set learning approach to few-shot.

To adequately probe how the prompt context *k* contributes to DSP performance, we further conduct subanalyses for few-shot learning to (1) examine the impact of increasing *k* by varying as 1, 5, 10 or 15 examples, and (2) compare the selection strategy of *k*.

#### 3.2.2. Application of GPT in Real-world DSP scenarios

##### 3.2.2.1. Cross-tissue generalization

In the within-tissue analysis, we fine-tune and test drug-cell line pairs originating from the same tissue type, assuming that the data distributions of the training and test sets are the same. However, for certain tissues (e.g., rare cancer), the cold-start problem is inevitable where cell lines are scarce and thus cannot suffice both the training and test sets. In such scenarios, it would be beneficial if the knowledge acquired during fine-tuning on a common tissue dataset could generalize to a rare tissue dataset. To mimic this real clinical setting where training and test samples are available for different tissues, in the cross-tissue analysis per dataset, we fine-tune the model using the largest tissue cohort in that dataset and subsequently apply the fine-tuned model to predict drug response on the remaining four tissue cohorts.

##### 3.2.2.2. Blind tests

In the standard experiment setup, the drug-cell line pairs are randomly split into training and test sets, so it is possible for the same drug or cell line to exist in both the training and test sets. This enables GPT-3 to predict the sensitivity of a new cell line to a drug, having already known the same drug’s effect on a different cell line during training, and vice versa. We conduct stringent splitting setups by constraining the drug or cell line from existing simultaneously in both the training and test sets. That is, (i) in the *blind test for cell line*, we predict the sensitivity of a new test cell line that is unseen in the training set to a test drug that is seen in the training set and (ii) in the *blind test for drug*, we predict the sensitivity of a test cell line that is seen in the training set to a new test drug that is unseen in the training set.

##### 3.2.2.3. Analysis of the drug-pathway associations

When a drug exerts its effects on a cell line, it is known to affect a related pathway rather than a single target (Wang et al. 2021). Henceforth, we leverage GPT-3’s pretrained text embeddings to generate the heatmap of Pearson correlations between the predicted drug responses (IC50) and pathway activity scores, with the possibility to obtain biological insights toward the drug response mechanism. More details are provided in Supplementary Note 7.

##### 3.2.2.4. Analysis of the top cell lines

In this case study, we want to investigate if GPT-3’s confidence of the drug response predictions (log probabilities) aligns with the reported experimental evidence in external databases. For illustrative purpose, we consider the cell lines of the drug Afatinib in the GDSC dataset as the test drug-cell line samples and evaluate them using the fine-tuned GPT-3 model. The log probabilities outputted by GPT-3 are sorted to find the cell lines most sensitive and resistant to Afatinib. To verify if the top cell lines predicted by GPT-3 are corroborated by relevant evidence, we use the online analysis portal (Fekete et al. 2022) which provides ranking of cell line models across four drug screening datasets. We consider Afatinib as it was evaluated in our benchmark dataset, GDSC, and was further validated in (Fekete et al. 2022).

#### 3.2.3 Baseline Comparisons

For baseline comparisons, we consider the following Transformer-based pretrained language models: *BERT* (Devlin et al. 2018), *BART* (Lewis et al. 2019), *BioBERT* (Lee et al. 2020), *DistilBERT* (Sanh et al. 2019), *RoBERTa* (Liu et al. 2019) and *ALBERT* (Lan et al. 2019). We also compare against three existing drug response models: *SWNet* (SWN) (Zuo et al. 2021), *PaccMann* (PM) (Manica et al. 2019) and *ConsDeepSignaling* (CDS) (Zhang et al. 2021). Please refer to Supplementary Note 8 for baseline descriptions and implementation details.

## 4. Results

**Figure 2:**
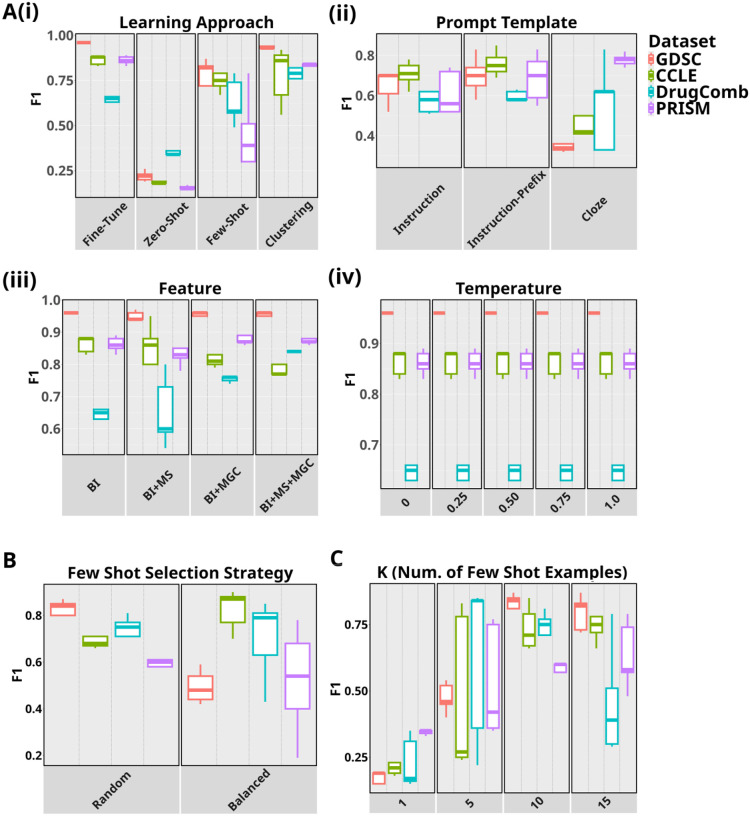
(A) Performance of GPT under different settings associated with the following methodological factors: (i) learning approach, (ii) prompt template, (iii) context and (iv) temperature. (B) Few-shot performance comparisons of datasets on varying the number of demonstrations, *k*, in increments of five from 1 to 15. (C) Performance comparisons between two different selection strategies under the few-shot setting.

**Figure 3:**
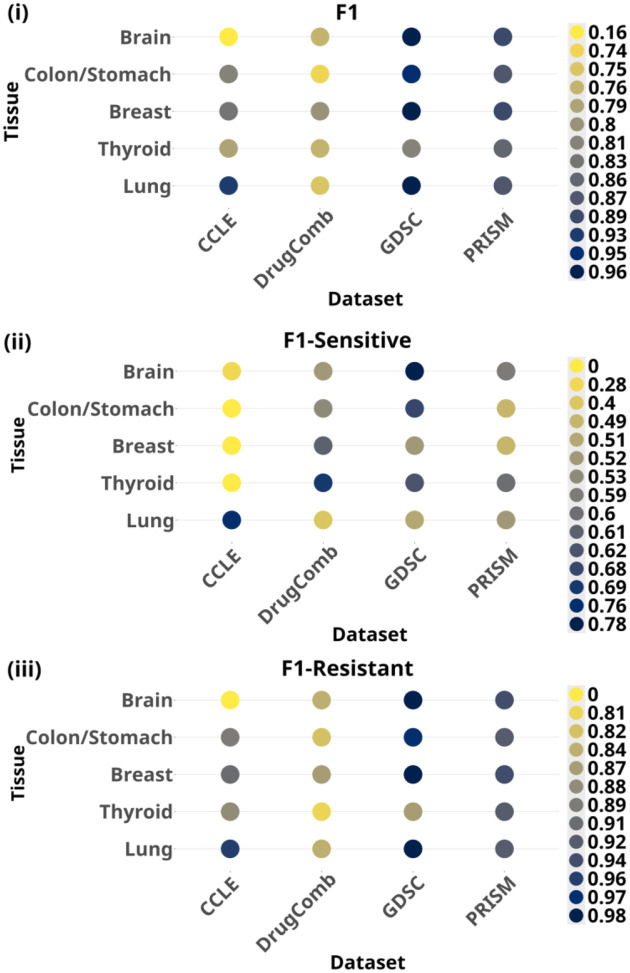
Performance of GPT with optimal settings on five tissue cohorts across different pharmacogenomics datasets evaluated with (i) F1 (ii) F1-Sensitive and (iii) F1-Resistant. F1 is the micro-averaged F1 score and F1-Sensitive and F1-Resistant are the F1 scores for the positive and negative classes, respectively. We used scikit-learn (Pedregosa et al. 2011) for the computation of evaluation metrics.

Figure 2A shows the evaluation results of the methodological factors over different datasets and tissues as box-plots. For ease of comparison among the settings within each factor, we consider the mean F1, which is computed by averaging the results pertaining to a setting across all the datasets and tissues. In the comparison of different learning approaches in Figure 2A(i) (detailed results in Supplementary Figure 4), fine-tuning attains the best performance with a mean F1 of 0.84 and provides significant improvements over zero-shot (0.24, p-value= 2.3e-18) and few-shot (0.66, p-value=1.5e-10), while rivaling with competitiveness against clustering (0.83, p-value=0.77). A comparison of the different prompt templates evaluated in the few-shot setting is presented in Figure 2A(ii) (detailed results in Supplementary Figure 5). We observe the best results with instruction-prefix (mean F1 0.68), resulting in performance gap of 4% against instruction prompt (mean F1 0.65), albeit a not statistically significant difference (p-value=0.40), and a more pronounced gap of 22% (p-value= 0.02) against cloze prompt (mean F1 0.55). We analyze the contribution of different combinations of features to the DSP performance in Figure 2A(iii) (detailed results in Supplementary Figure 6). We use performance with BI as a reference point to evaluate the change in performance instigated by the three feature sets. Overall, incorporating the drug’s molecular representation in the SMILES notation (i.e., BI + MS) does not provide any added predictive power, but rather causes a 3% decrease (p-value=0.46) of the mean F1 performance from 0.84 to 0.81. With regard to the BI + MGC feature, prompt context derived from drug’s synergy information in DrugComb dataset and drug’s mechanism of action in PRISM lead to performance boosts (13.4% mean F1 increase, p-value=0.02 and 2.3% mean F1 increase, p-value=0.28, respectively). Gene mutation information in GDSC does not cause any drastic change in performance and, conversely, gene expression in the CCLE dataset has a negative effect (3.5% mean F1 decrease; p-value=0.31). Lastly, contextualizing the prompt with both MGC and SMILES (i.e., BI + MS + MGC) generally lags behind in mean F1 performance in comparison to the absence of SMILES (BI + MGC) by 1% (p-value=0.72). In the temperature analysis (Figure 2A(iv)), the performance remains unaffected across the different settings. Based on the best evaluation results of the methodological factors, we test GPT-3’s DSP capability under the optimal methodological settings on the five tissues cohorts across the datasets. The F1 and per-class F1 results in Figure 3 suggest that GPT-3 performed competitively on the GDSC and PRISM datasets across all the tissues, while the worst on the CCLE dataset with performance declines in F1 (p-value=0.15), F1-Sensitive (p-value=0.02) and F1-Resistant (p-value=0.24).

Comparison of performances with different number of demonstration examples *k* in Figure 2B reveals that GPT-3’s in-context generalizability is sensitive to *k* and prompting with more drug-cell line examples (i.e., higher *k*), in general, is beneficial. The experiments comparing between the random and balanced selection strategies of *k* in Figure 2C provide mixed results across datasets. Balanced selection strategy surpasses random selection in CCLE and PRISM, while the reverse is true for GDSC and DrugComb, although neither of them is statistically significant.

**Figure 4:**
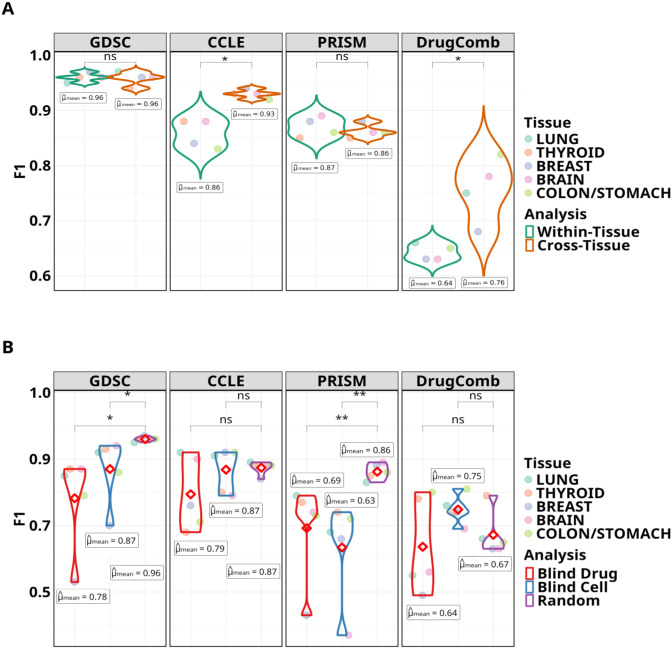
Performance comparisons in the form of violin plots for the (A) analysis between within-tissue and cross-tissue evaluation settings and (B) blind test analyses for drugs and cell lines. In each type of analysis, GPT is evaluated on the five tissue cohorts associated with each dataset separately. So, the annotated mean (also shown as the red diamond shape) is computed by averaging the results across all tissues within a dataset. The degree of statistical significance is indicated by the number of asterisks and a not statistically significant difference by ‘ns’.

We compare GPT-3’s within-tissue and cross-tissue prediction abilities across the tissues within each dataset as violin plots in Figure 4A. In GDSC and PRISM evaluations, the cross-tissue performances are able to uphold comparable results to the corresponding evaluations in the within-tissue setting. Whereas evaluations on the CCLE and DrugComb datasets attain heightened cross-tissue performances with significant mean F1 performance gains of 8.1% (p-value=0.001) and 18.7% (p-value=0.009), respectively.

The violin plots in Figure 4B depict GPT-3’s blind test generalizations (Blind Drug, Blind Cell) over the five tissue cohorts within each dataset. The baseline performance was established by evaluation on the random test split (Random). GPT-3 was able to attain competitive performances (as not statistically significant difference) on par with the corresponding random evaluations for CCLE and DrugComb. In contrast, the blind evaluations underperform on PRISM (p-value=0.01) and GDSC (p-value=0.05). Comparing between blind drug and blind cell evaluations, generally, GPT-3 lags behind in accurately predicting response of drug-cell line pairs that include new drugs, with mean F1 performance gains toward blind cell tests for the following datasets: GDSC (11.53%; p-value=0.29), CCLE (10.12%; p-value=0.23) and DrugComb (17.1%; p-value=0.13).

**Figure 5:**
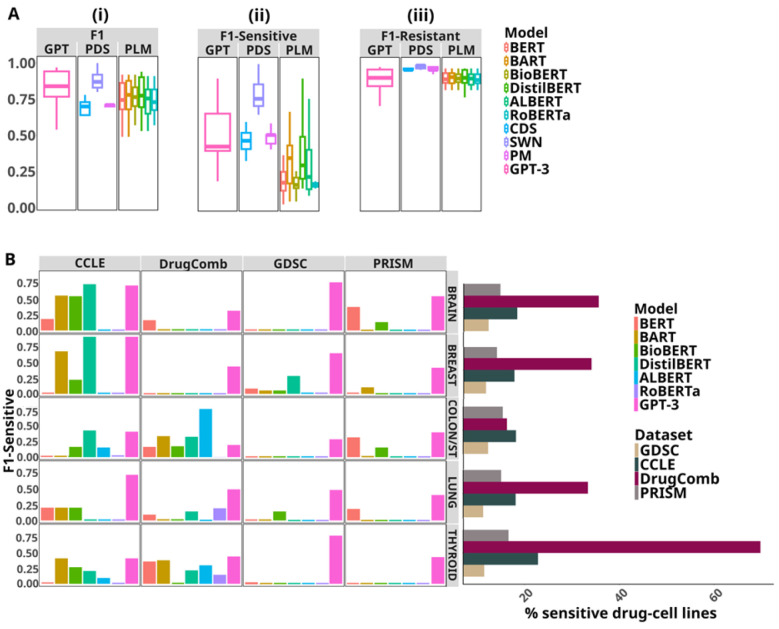
(A) Performance comparisons of GPT with two types of baseline models, previous drug response prediction models (PDS) and pretrained language models (PLM). GPT and the PLM baselines are fine-tuned. The F1 and per-class F1 reported are averaged across all five tissues’ evaluations across the four datasets for each model. Detailed results are available in Supplementary Figure 7 and Supplementary Figure 8. (B) F1-Sensitive performances of GPT and baselines with varying positive class distributions.

In Figure 5A (detailed results in Supplementary Figures 7 and 8), we compare the F1 and per-class performances of GPT-3 with baseline models. GPT-3 outperforms baselines with mean F1 gains of 5.4% in GDSC, 13.6% in CCLE, 5.3% in DrugComb and 5.3% in PRISM (p-values reported in Supplementary Note 9). Additionally, GPT-3 was able to maintain superior F1-Sensitive performance against BERT (p-value=3.6e-05), BART (p-value=0.05), BioBERT (p-value=0.00026), DistilBERT (p-value=0.15), ALBERT (p-value=0.13), RoBERTa (p-value=0.03), CDS (p-value=0.97) and PM (p-value=0.012). Although SWN’s F1 and F1-Sensitive scores surpass GPT-3’s, the differences are not statistically significant (p-value=0.06 and p-value=0.59 respectively). In Figure 5B, we examine the F1-Sensitive performances of GPT and baselines with varying positive class distributions. In a label scarce setting with only 20% of sensitive drug-cell lines available in GDSC’s tissue cohorts, GPT-3 is seen to perform significantly more robustly compared to the baselines (p-value=1.1e-22).

**Figure 6:**
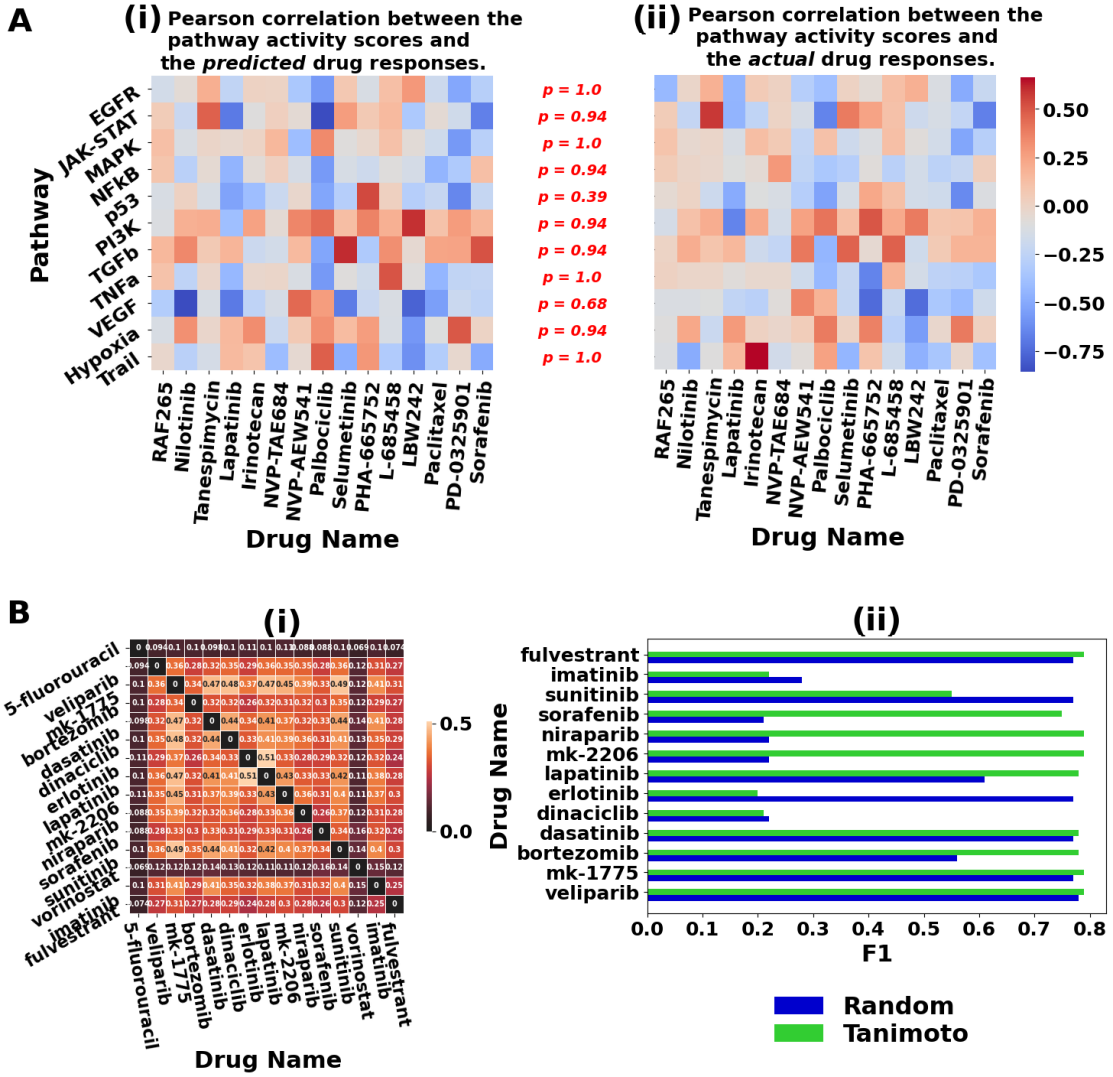
(A) Drug-pathway associations in the CCLE dataset. Negative (blue) and positive (red) correlations correspond to ‘assistant’ and ‘resistant’ associations, respectively. The p-values, annotated in red (i.e., p =), are computed using the Kolmogorov-Smirnov test and indicate distribution similarity between the predicted (subplot (ii)) and actual (subplot (ii)) drug-pathway associations. Note that the null hypothesis (p > 0.05) is that the two distributions are similar. (B (i)) Heatmap visualization of Tanimoto correlations between drugs. (ii) Few-shot classification performance using Tanimoto-based (i.e., structurally similar drugs) selection for in-context examples in the prompt.

We visualize heatmap of the Pearson correlations between the predicted drug responses (IC50) and pathway activity scores of the cell lines generated by PROGENy (Schubert et al. 2018) in Figure 6A. The computed p-values assert high similarity between the corresponding correlation distributions (i.e., predicted (Figure 6A(i)), actual (Figure 6A(ii))) across all pathways, thus justifying that GPT-3-derived text embeddings could facilitate in both the accurate prediction and explainability of the drug response. Importantly, we observed that the drugs with relatively low IC50 scores were sensitive to major pathways and these findings align with those in the previous research (Section 5). In a case study analysis, we further corroborate GPT-3’s generalization in terms of its predicted log probabilities for the drug Afatinib by ranking its top cell lines in Supplementary Table 3. GPT-3 was able to predict drug response with high confidence for the most sensitive and resistant cell lines to the drug Afatinib, which have been validated in previous experimental studies (Fekete et al. 2022).

## 5. Conclusion and Discussion

The advent of LLMs have revolutionized the field of natural language processing. However, their applicability to biomedical tasks with structured data is still missing or limited in the current research. This work showcases the feasibility and efficacy of a generative LLM, namely GPT-3, in the prediction of cancer cell line’s response to oncology and non-oncology drugs, with a focus on leveraging structured pharmacogenomics data. To account for the crucial role prompt engineering plays in LLM’s performance, this study provides a nuanced exploration of methodological factors in tailoring prompt engineering to optimize the DSP task. Additionally, it contributes valuable insights toward GPT-3’s application in DSP that cater to diverse real-world evaluation scenarios, paving the way for personalized treatment.

We investigated the effect of the type of feature integrated into the prompt context on GPT-3’s performance. The results suggest that drug descriptor in the form of SMILES can be, in general, detrimental from GPT-3’s generalization perspective. The SMILES notation linearly represents the atom connectivity in a chemical compound as a text sequence. However, unlike a natural language text, the characters in a SMILES sequence correspond to topological characteristics, such as ring-closure or branches, that cannot be treated as isolated entities. Furthermore, the presence of repetitive tokens in the SMILES string causes the atoms of a molecule to be indistinguishable in the token space (Ucak et al. 2023). We reason that the drop in performance was as a result of GPT-3’s failure in extracting such nuanced features from the SMILES-incorporated prompt. This is likely attributed to GPT-3’s base vocabulary not accounting for SMILES grammar, that hinders the frequency-based Byte Pair Encoding tokenization (Sennrich et al. 2015) from effectively splitting the SMILES sequence into meaningful constituent elements without losing the molecule’s chemical validity. Unsupervised pretraining of GPT-3 using a large SMILES corpus could possibly warrant performance enhancement. In contrast, supplementing the drug-cell line input with drug’s functional feature aids in performance improvements. In relation to DrugComb, drug synergy has shown to have a positive correlation with drug sensitivity such that cell lines involved in synergistic drug combination are also significantly more sensitive to the drugs (Narayan et al. 2020). Similarly, in PRISM evaluation, drug sensitivity is affected by the drug’s MOA as it identifies the binding target and whether the therapeutic effects will be enabled by the activationor inhibition of the target’s function. Although genomic mutations are deemed to provide significantly discriminative patterns across cell lines (Yang et al. 2012), it only benefitted the DSP performance on GDSC’s lung cohort evaluation. The use of gene expression feature was dominated by a decline in CCLE’s performances across tissues. We rationalize CCLE’s drop in performance to the “small n, large p” problem (Nguyen et al. 2021) wherein given the smaller number of drug-cell line pairs in the dataset, the gene expression sequence increases the overall feature dimensionality (i.e., prompt context length) causing obstruction in the model’s learning and resulting in possible overfitting.

We examined GPT-3’s DSP capability through a diverse spectrum of learning approaches. Zero-shot learning is a training-free approach that generates the drug response by conditioning on the input prompt and relying on the knowledge already encoded in GPT-3’s parameters. The unfavorable zero-shot generalization of GPT-3 suggests that it was not exposed to sufficient pharmacogenomics-related content during its pretraining phase. However, augmenting the prompt with a few drug-cell line examples via few-shot prompting, that demonstrate the input-output mapping existing in the drug sensitivity datasets, initiates relevant learning and hence is seen to elevate the overall performances. This is consistent with the findings in the general domain where GPT-3 excelled in NLP tasks in the few-shot setting (Brown et al. 2020). Our analysis of prompt engineering in the few-shot setting suggests that providing explicit instruction and conforming to a concise in-context exemplar format (i.e., instruction-prefix prompt) elicits the most accurate drug response prediction from GPT-3. Drugs with similar chemical structure have shown to exhibit similar sensitivity patterns (Zhang et al. 2015). We emulate this finding in GPT-3’s in-context learning (Supplementary Note 10) and observe that demonstration examples selected based on structurally similar drugs enhance GPT-3’s few-shot classification, in general (Figure 6B). Fine-tuning entails training GPT-3 on supervised pharmacogenomics datasets. The domain-specific knowledge acquired thereafter visibly causes substantial spike in the performances of all datasets. In the weakly supervised paradigm, we iteratively refine GPT-3’s pretrained embeddings by clustering via BGMM. This benefits GPT-3’s DSP generalization in comparison to inference contingent on its pre-existing knowledge (zero-shot), resulting in significant performance gains (mean F1 increase from 0.24 to 0.83). More importantly, given that the pseudo-labels for cluster initialization are derived from fine-tuning, their mean F1 performances are comparable and at times clustering even exceeds fine-tuning performance, exemplified in the following evaluations: DrugComb’s lung, thyroid, breast, brain and CCLE’s breast.

GPT-3 has demonstrated remarkable out-of-distribution generalization, underscoring its ability to predict drug response for cancer types completely unobserved during training. The cross-tissue evaluation corresponds to simulating the challenging cold-start scenario in precision oncology, where a large training dataset is not readily available for certain tissue types but only a few samples could be exploited for testing. Harnessing transfer learning that relies on fine-tuning GPT-3 on a large amount of common tissue drug response data has potential use case in recommending treatments to patients with rare cancers. We further evaluated GPT-3 in challenging data splitting strategies. The blind cell test aims to find candidate drugs among all the drugs the model is trained on for a new cell line (i.e., new patient) and has direct utility in personalized cancer treatment. The blind drug test, on the other hand, is designed to generalize for a new drug to known cell lines and could be useful in repurposing non-cancer drugs for cancer treatment, as well as novel drug discovery (Partin et al. 2023). Our empirical results suggest that GPT-3’s prediction performances under the stricter blind partitioning settings are comparable to those with the baseline random splitting on CCLE and DrugComb. We further observed that GPT-3’s generalization in the blind cell analysis is generally superior to the blind drug test, although the difference is not statistically significant. Even though blind cell analysis ensures that the test set does not contain any cell lines already present in the training set, it is possible to share biological characteristics if the unknown test cell lines and known train cell lines happen to belong to the same tissue. On the contrary, the relatively large chemical space of drug compounds makes it difficult for GPT-3 to learn generalizable features for an unknown drug.

We benchmarked GPT-3 against a range of established baseline models for a rigorous performance assessment on the drug sensitivity prediction task. GPT-3 outperformed the baselines by achieving a higher mean overall F1 score, with mostly statistically significant performance differences for evaluations on the GDSC, CCLE and PRISM datasets. Importantly, F1-Sensitive is the most relevant metric for drug response prediction as it shows the correctly classified sensitive drug-cell line pairs, knowledge of which could accelerate treatment optimization and drug repurposing. On inspecting the Transformer-based language model baseline generalizations for the sensitive class, there are some instances where GPT-3’s overall F1 score is below the baseline performances (e.g., thyroid assessment in CCLE, brain assessment in DrugComb), however, it is procured at the expense of relatively lower corresponding F1-Sensitive scores, in general. This indicates that GPT-3 is not only able to successfully distinguish the sensitive samples from the resistant ones, but also strikes a good balance in accurately identifying drug-cell line pairs belonging to the minority sensitive class. This could be attributed to GPT-3’s larger size (175B parameters) and the fact that it has been pretrained on much more data (300B tokens), that helps to encapsulate domain-specific signals better during fine-tuning. In comparison to the existing drug response models (i.e., CDS, SWN, PM), GPT-3’s superior performance implicates the predictive benefit of prompt-based input engineering over the traditional feature engineering in providing task-specific context. The superimposition of sensitive class distributions of tissues across the datasets with F1-Sensitive performances (Figure 5B) further indicates that GPT’3’s fine-tuning capability for DSP is not constrained by label scarcity as performances on thyroid, breast, brain and colon/stomach tissues of GDSC, which has the fewest sensitive samples, supersede counterpart cohorts on DrugComb with the largest positive class distributions.

In order to accommodate GPT-3 for interpretability study, we conducted a post-hoc analysis based on drug-pathway associations through leveraging GPT-3’s pretrained text embeddings of input prompts. As a drug is known to exert its effects by influencing the gene expression in the related pathway (Wang et al. 2021), the drug-pathway associations facilitate in providing biological justifications for the predicted drug responses. We observed several instances of drug-pathway associations for which our calculated Pearson correlations are consistent with the findings in previous research. Sorafenib is reported to inhibit the tumor cell growth by blocking the MAPK pathway, indicating an assistant association (Gauthier et al. 2013). Sorafenib, a multi-kinase inhibitor, and RAF265, a RAF inhibitor drug (McCubrey et al. 2007), are found to be negatively correlated (assistant association) with the VEGF pathway (Liu et al. 2006). Analogously, Nilotinib, a tyrosine kinase inhibitor (TKI), is known to alter VEGF-induced angiogenesis pathway (Zibrova et al. 2024). We observed negative correlation between the EGFR pathway and Lapatinib, which is an EGFR inhibitor (Montemurro et al. 2007). The drug LBW242 is known to sensitize cancer cells to the antitumor effects of TRAIL (Philemon et al. 2015), indicating an assistant association.

Despite the promising performances, the safety-critical nature of the domain necessitates that some limitations need to be addressed before a generative LLM like GPT-3 can be deployed and integrated into the medical workflow. In the present study, we tested GPT-3’s interpretability by leveraging its pretrained text embeddings. A possible future direction would be to directly prompt GPT-3 via the few-shot chain-of-thought strategy (Wei et al. 2022) with the possibility to generate the reasoning chains justifying its predicted response, while incorporating human-in-the-loop to correct any reasoning errors or hallucinations. Considering the closed-source nature of GPT-3 family models’ accessibility, we anticipate future efforts of applying the systematic methodology presented in this work to investigate open-source LLMs (e.g., LLaMA) for a broader understanding of DSP evaluation.

## Supplementary Material

1
