# Supplementary material for: SensitiveCancerGPT: Leveraging Generative Large Language Model on Structured Omics Data to Optimize Drug Sensitivity Prediction
Source: bioRxiv. 2025 Mar 3:2025.02.27.640661. Preprint. [Version 3] doi: 10.1101/2025.02.27.640661 (PMC11888479; doi:10.1101/2025.02.27.640661)
Supplement: 1 [file NIHPP2025.02.27.640661V3-supplement-1.pdf]

bioRxiv preprint doi: https://doi.org/10.1101/2025.02.27.640661; this version posted March 3, 2025. The copyright holder for this preprint (which was not certified by peer review) is the author/funder, who has granted bioRxiv a license to display the preprint in perpetuity. It is made available under aCC-BY 4.0 International license.

Supplementary Material

Supplementary Note 1: Background

Computational Drug Sensitivity Prediction

The various methods employed to predict drug response in silico can be categorized into network-based approaches (Guan et al. 2019, Cheng et al. 2019) and machine learning approaches (Dong et al. 2015, Menden et al. 2013). Network-based approaches model the relationships between the heterogeneous entities in the pharmacogenomic data (e.g., drug, target, cell line) by constructing similarity or interaction graphs. Machine learning approaches, on the other hand, extract informative features from the pharmacogenomics data, based on which training and prediction of drug sensitivity is carried out. Recently, deep learning has rapidly made strides owing to its ability to automatically learn the complex, non-linear patterns from large amounts of pharmacological and cell line omics data (Baptista et al. 2021, Stephenson et al. 2019, G et al. 2020). Importantly, effective representations are learned by encoding chemical and genomic properties that could facilitate the automatic extraction of latent features informative in the drug response prediction. DeepDSC (Li et al. 2019) first learns compressed representation of gene expression data through the application of a stacked autoencoder, which is then integrated with drug chemical features in the form of molecular fingerprints to predict drug response using a feed forward neural network. DeepDR (Chiu et al. 2019) employs separate autoencoders pretrained on pan-cancer data to transform gene mutation and expression features into low dimensional representations and links them for drug response prediction. Unlike autoencoder-based approach, end-to-end drug response models have also been proposed that jointly encode and predict as a unified model. SWNet (Zuo et al. 2021) adopts a dual convergence model architecture to simultaneously encode the drug and genomic features using graph neural network and convolutional neural network, respectively. It then exploits late integration between the two representations before passing through the prediction subnetwork. Manica et al. 2019 proposes a multimodal architecture and explores various models for encoding drug representation from SMILES, while using an attention-based gene encoder.

This work explores the application of a generative LLM (i.e., GPT) in drug sensitivity prediction (DSP). Unlike standard deep learning models which focus on automatic feature engineering from the data for optimization on downstream tasks, GPT-based LLMs exploit input engineering that leverages textual prompts to reformulate downstream tasks through the incorporation of task-specific details.

Generative LLM for biomedical tasks

The proliferation of biomedical corpora and the idiosyncrasies of biomedical text have led to domain-specific research of LLM in biomedicine. Gutierrez et al. 2022 explored the in-context learning capability of GPT-3 for named entity recognition and relation extraction. Moradi et al. 2021 also evaluated GPT-3 in the few-shot setting but across a broader range of biomedical NLP tasks encompassing textual inference, relation classification, semantic similarity estimation, question answering and text classification. Labrak et al. 2023 performed a comprehensive assessment of four instruction-tuned LLMs (ChatGPT, Flan-T5 UL2, TkInstruct, and Alpaca) in the zero-shot and few-shot settings on 13 biomedical tasks covering diverse NLP problems: classification, question answering, relation extraction, natural language inference and named entity recognition.
However, these studies focus on processing NLP datasets that include unstructured text and their applicability to biomedical tasks with structured data is still missing or limited in the current research.

bioRxiv preprint doi: https://doi.org/10.1101/2025.02.27.640661; this version posted March 3, 2025. The copyright holder for this preprint (which was not certified by peer review) is the author/funder, who has granted bioRxiv a license to display the preprint in perpetuity. It is made available under aCC-BY 4.0 International license.

Given that structured pharmacogenomics data have special data formats that a generative LLM is not naturally familiar with and cannot well comprehend as it is pretrained on textual Web data, specialized prompt design that involves linearizing the structured input into sentence to elicit the most accurate drug response is non-trivial. This work aims to bridge this research gap by introducing prompt engineering that is tailored to structured pharmacogenomics data to facilitate DSP.

Deep neural networks for tabular data

Tabular data is ubiquitous in many real-world domains, including healthcare (Bhatt et al. 2020, Jiang et al. 2024). Data within a table is laid out in rows and column format, with each row corresponding to an input sample and each column representing a feature attribute. Thus, accurate prediction over tabular data necessitates semantic structure understanding capabilities. The wide application of deep neural networks to tabular data is attributed to its ability in automatically learning non-linear feature representations from table cells. Several variants of the vanilla multilayer perceptron (MLP) have demonstrated improved performance on tabular data. Work by Kadra et al. 2021 applied several regularization techniques (i.e., regularization cocktail) to the MLP, a parameter-efficient ensemble of MLP was proposed in Gorishniy et al. 2024, and an enhanced MLP obtained through better default parameters was presented in Holzmüller et al. 2024. Inspired by the remarkable performance of decision trees ensemble methods on tabular data (Shavitt et al. 2018, Grinsztajn et al. 2022, McElfresh et al. 2024), studies have attempted to implement neural network-based decision forest by ensuring differentiability (Popov et at. 2019, Marton et al. 2024). More recently, neural networks built upon the self-attention mechanism of Transformer have been proposed (Padhi et al. 2021, Gorishniy et al. 2021, Hollmann et al. 2022, Huang et al. 2020 and Arik et al. 2021).

However, given that deep learning has also shown to fare poorly with sub-par performance on structured tabular data (Borisov et al. 2022, Shwartz-Ziv et al. 2022), it is not well suited for omics data. In this work, we propose domain-specific prompt engineering of a generative LLM to linearize tabular pharmacogenomics data. Consequently, we were able to outperform the performances of several state-of-the-art deep learning baselines on the DSP task.

bioRxiv preprint doi: https://doi.org/10.1101/2025.02.27.640661; this version posted March 3, 2025. The copyright holder for this preprint (which was not certified by peer review) is the author/funder, who has granted bioRxiv a license to display the preprint in perpetuity. It is made available under aCC-BY 4.0 International license.

Supplementary Note 2: Descriptions of Pharmacogenomics Datasets

GDSC: The Genomics of Drugs Sensitivity in Cancer (GDSC) (Yang et al. 2012) is an open-source database consisting of screening response data of tumoral cell lines to anticancer treatments. In phase 1 (GDSC1), the sensitivity of 987 cancer cell lines to 320 compounds were assayed, while phase 2 experiment (GDSC2) assayed an additional 809 cancer cell lines to 175 compounds (with some overlapping samples in the GDSC1 assay). Cancer cell lines are characterized by genetic features, such as the mutation state. We downloaded the raw dose-response data for GDSC2 from http://www.cancerrxgene.org/downloads/.

CCLE: The Cancer cell line Encyclopedia (CCLE) (Barretina et al. 2012) contains a large-scale genomic data (e.g., gene expression) obtained using Affymetrix U133 + 2 arrays for 947 human cancer cell lines and response data for around 500 of the cell lines to 24 drug compounds across 36 tumor types. There are 491 common cancer cell lines having both drug sensitivity measurements and gene expression profile data. The data were downloaded from the CCLE website (http://www.broadinstitute.org/ccle) and PharmacoGx R package.

DrugComb: The DrugComb (Zagidullin et al. 2019, Zheng et al. 2021) dataset includes data on synergy and sensitivity of drug combinations. It also includes single drug sensitivity that is characterized as a dose-response curve in terms of IC50 with 717,684 single drug screenings from 37 studies (March 2021). For each drug-drug sample in the dataset, we pair each drug with the cell line to form two separate drug-cell line pairs and include the corresponding single drug IC50 scores. The data was downloaded from https://drugcomb.org/.

PRISM: The secondary PRISM Repurposing dataset (Corsello et al. 2020) is a drug repurposing database that includes results of pooled-cell line chemical-perturbation viability screens for 1448 drug compounds screened against 499 cell lines. The original clinical indications for majority (53%) of the active compounds were for non-oncology purposes. The data was downloaded from https://depmap.org/portal.

bioRxiv preprint doi: https://doi.org/10.1101/2025.02.27.640661; this version posted March 3, 2025. The copyright holder for this preprint (which was not certified by peer review) is the author/funder, who has granted bioRxiv a license to display the preprint in perpetuity. It is made available under aCC-BY 4.0 International license.

Supplementary Note 3: Cancer Cohorts

We focus on the following five cancer tissue types in our evaluation - *lung*, *thyroid*, *breast*, *brain*, *colon/stomach* - as they are available across all four datasets (GDSC, CCLE, DrugComb, PRISM) for evaluation. Note that the aforementioned tissue names could be different in some datasets (e.g., brain, central nervous system). We removed the drug-cell line pairs with missing drug or cell line from each cohort dataset which resulted in the dataset sizes shown in **Supplementary Table 1**. For training and testing GPT, we randomly divided each dataset with an 80%-20% stratified split. Note that for GDSC, as the class distributions are imbalanced with fewer drug-cell line pairs available for the sensitive class (<15%), as shown in **Supplementary Table 2**, we oversampled the minority class (i.e., sensitive) in the training sets of the tissues in GDSC.

bioRxiv preprint doi: https://doi.org/10.1101/2025.02.27.640661; this version posted March 3, 2025. The copyright holder for this preprint (which was not certified by peer review) is the author/funder, who has granted bioRxiv a license to display the preprint in perpetuity. It is made available under aCC-BY 4.0 International license.

Supplementary Note 4: Comparative Evaluation between GPT-3, GPT-3.5, GPT-4

We carried out fine-tuning analyses on CCLE's tissue cohorts using the GPT-3, GPT-3.5 and GPT-4 models. This entailed sending a fine-tuning job request to OpenAI's Completions API for GPT-3. For GPT-3.5 and GPT-4, we sent the respective fine-tuning job requests to OpenAI's Chat Completions API, with each drug-cell line input in the dataset formatted as a conversation (**Supplementary Figure 1**). We found that GPT-3.5 and GPT-4 performed the same as GPT-3 with no additional improvements; hence this positions GPT-3 as a more cost-effective option for drug sensitivity prediction.

bioRxiv preprint doi: https://doi.org/10.1101/2025.02.27.640661; this version posted March 3, 2025. The copyright holder for this preprint (which was not certified by peer review) is the author/funder, who has granted bioRxiv a license to display the preprint in perpetuity. It is made available under aCC-BY 4.0 International license.

Supplementary Note 5: Learning Approach

We employ four different paradigms to adapt GPT-3 for downstream drug sensitivity prediction. (i) *Fine-tuning* is based on supervised learning where the pretrained GPT-3 model is trained on prompt-completion pairs in the training set for 4 epochs and its performance is evaluated on the test set. (ii) *Zero-shot* relies on the unsupervised pretrained GPT-3 model's parametric knowledge to generate drug sensitivity response during inference. This is facilitated by inputting textual prompt that includes the test sample and task-specific description. (iii) *Few-shot* is similar to zero-shot but also inserts a handful of demonstration examples ("shots") into the prompt as domain-specific knowledge to learn from, referred to as in-context learning. For zero-shot and few-shot experiments, we applied GPT-3 only on the test split for evaluation. (iv) We introduce a weakly supervised learning approach, *clustering embeddings* (Fei et al. 2022), that utilizes text embeddings. These embeddings are first retrieved from OpenAI's embedding API by feeding drug-cell line prompts as the input. The retrieved embeddings are then clustered using a Bayesian Gaussian Mixture Model (BGMM) (Bishop C 2006). The clusters are initialized using pseudo-labels derived from the fine-tuning predictions and iteratively refined using BGMM. We compare the final cluster assignments for the test samples against their ground truths for evaluation.

We performed each evaluation 3 times and considered the average as the final F1 score.

bioRxiv preprint doi: https://doi.org/10.1101/2025.02.27.640661; this version posted March 3, 2025. The copyright holder for this preprint (which was not certified by peer review) is the author/funder, who has granted bioRxiv a license to display the preprint in perpetuity. It is made available under aCC-BY 4.0 International license.

Supplementary Note 6: Feature Descriptions

The first feature is the drug's molecular structure information (MS) in the simplified molecular input line entry specification (SMILES) format (Weininger D. 1998). We use additional molecular or genomic context (MGC) as the second feature and this feature varies across the datasets due to the lack of another common feature among all four datasets. In particular, it includes *gene mutation* in segment for GDSC, *gene expression* for CCLE, *drug-drug synergy* information for DrugComb and *drug's mechanism of action* (*MOA*) for PRISM. For CCLE, the gene expression data of a cell line is available for about 20,000 genes, corresponding to a vector of the same length. As a result of the high dimensionality overhead of the gene expression data, we sort the gene vector for each cell line by their transcript level values in descending order and select the top 200 genes to represent the gene expression of the cell line as the MGC feature. For DrugComb, we use a Loewe score greater than 5 to categorize the drug-drug synergy information as either 'synergistic' or 'not synergistic' (Li et al. 2023).

Examples of input prompt templates with MGC are depicted in **Supplementary Figures 3A-3D** for the four datasets. These two features are integrated with the drug-cell line input pair (Basic Information (BI)), leading to three feature groups in total (BI + MS, BI + MGC, BI + MS + MGC), that are evaluated per dataset.

bioRxiv preprint doi: https://doi.org/10.1101/2025.02.27.640661; this version posted March 3, 2025. The copyright holder for this preprint (which was not certified by peer review) is the author/funder, who has granted bioRxiv a license to display the preprint in perpetuity. It is made available under aCC-BY 4.0 International license.

Supplementary Note 7: Analysis of the drug-pathway associations

When a drug exerts its effects on a cell line, it is known to affect a related pathway rather than a single target (Wang et al. 2021). Henceforth, to evaluate the interpretability of GPT-3, we identify the associations between drugs and signaling pathways, with the possibility to obtain biological insights. In order to infer the drug-pathway associations, we generate the heatmap of Pearson correlations between the predicted drug responses (IC50) and pathway activity scores. However, as large language models such as GPT-3 are naturally suited for classification rather than regression problems, we could not use GPT-3 to predict the IC50 scores. So as a work around, we leverage the embedding model available through OpenAI's embedding API (https://api.openai.com/v1/embeddings) to retrieve text embeddings of the input prompts in the CCLE dataset. The input prompts include the drug name, cell line name and top 200 genes in the gene expression from the CCLE dataset. We then train a Random Forest model using the GPT-3-derived embeddings as the input features and optimize it to predict the IC50 score. While to calculate the pathway activity scores, we consider the following 11 cancer-relevant pathways from PROGENy (Schubert et al. 2018): *EGFR*, *MAPK*, *PI3K*, *VEGF*, *JAK-STAT*, *TGFb*, *TNFa*, *NFkB*, *Hypoxia*, *p53-mediated DNA damage response*, and *Trail*. Subsequently, PROGENy is applied to the gene expression data from the CCLE dataset to calculate the pathway activity score of each cell line.

In the computed Pearson correlations between the predicted drug responses (IC50) and pathway activity scores of the cell lines (**Figure 6A(i)**), a negative correlation indicates that the drug inhibits the pathway by reducing the expression of genes in the pathway (i.e., assistant), while a positive correlation denotes pathway activation induced by the drug via increasing the expression of genes in the pathway (i.e., resistant) (Wang et al. 2021). These inferred drug-pathway associations are compared side by side to the actual drug-pathway associations (**Figure 6A(ii)**) - obtained by computing Pearson correlations in relation to the ground truth drug responses in the CCLE dataset. We quantify the extent of similarity between the two distributions by performing the Kolmogorov-Smirnov test between the predicted and actual Pearson correlations. The p-values measured by the test based on the null hypothesis that the two distributions are identical ($p\text{-value} > 0.05$) are annotated in red in **Figure 6A**.

bioRxiv preprint doi: https://doi.org/10.1101/2025.02.27.640661; this version posted March 3, 2025. The copyright holder for this preprint (which was not certified by peer review) is the author/funder, who has granted bioRxiv a license to display the preprint in perpetuity. It is made available under aCC-BY 4.0 International license.

Supplementary Note 8: Baseline Models

For baseline comparisons, we consider the following Transformer-based pretrained language models: (i) *BERT* (Devlin et al. 2018) is a masked language model that leverages Transformer's encoder; (ii) *BART* (Lewis et al. 2019) is a generative language model that leverages a BERT-like encoder and GPT-like decoder; (iii) *BioBERT* (Lee et al. 2020) is the domain-specific variant of BERT pretrained on large biomedical corpora. (iv) *DistilBERT* (Sanh et al. 2019) is a smaller version of BERT that leverages knowledge distillation during pretraining; (v) *RoBERTa* (Liu et al. 2019) is similar to BERT but is pretrained using only masked language modeling objective with different hyperparameters and (vi) *ALBERT* (Lan et al. 2019) is a parameter-efficient version of BERT enabled through factorized embedding parameterization and parameter sharing across layers. We used the models provided by HuggingFace (https://huggingface.co/). All models are fine-tuned on the training set for four epochs and evaluated on the test set. We also compare against three existing drug response models: (i) *SWNet* (SWN) (Zuo et al. 2021) adopts a dual convergence model architecture to simultaneously encode the drug and genomic features using graph neural network and convolutional neural network, respectively. It then exploits late integration between the two representations before passing through the prediction subnetwork; (ii) *PaccMann* (PM) (Manica et al. 2019) proposes a multimodal architecture and explores various models for encoding drug representation from SMILES, while using an attention-based gene encoder and (iii) *ConsDeepSignaling* (CDS) (Zhang et al. 2021) integrates signaling pathway information with genomic features through a gene-pathway connection matrix and trains a deep belief network. As these three models were optimized for IC50 prediction in the published works, we tweaked the models' implementations (downloaded from the respective GitHub repository) for reformulation as a binary classification task to maintain a fair comparative analysis with GPT-3. We evaluated these three models on the GDSC dataset.

bioRxiv preprint doi: https://doi.org/10.1101/2025.02.27.640661; this version posted March 3, 2025. The copyright holder for this preprint (which was not certified by peer review) is the author/funder, who has granted bioRxiv a license to display the preprint in perpetuity. It is made available under aCC-BY 4.0 International license.

Supplementary Note 9: Baseline Comparisons

On the GDSC dataset, GPT-3 asserted superior F1 performance over ALBERT, BART, BERT, BioBERT, DistilBERT, RoBERTa, CDS, SWN, PM with mean performance gains over all tissues of 5.6% (p-value=5e-05), 5.4% (p-value=5e-05), 5.4% (p-value=9e-05), 5.2% (p-value=0.0001), 5.2% (p-value=2.2e-05) and 5.6% (p-value=2.2e-05), 43% (p-value= 0.0004), 9% (p-value=0.06) and 68% (p-value=0.02), respectively. GPT-3's performance improvements on the other datasets are as follows: CCLE dataset - ALBERT (22%; p-value=0.02), BART (8.7%; p-value=0.24), BERT (16%; p-value=0.09), BioBERT (12%; p-value=0.01), DistilBERT (5.2%; p-value=0.008), and RoBERTa (18%; p-value=0.009); DrugComb dataset - ALBERT (10%; p-value=0.91), BART (3.6%; p-value=0.73), BERT (0.73%; p-value=0.53), BioBERT (7.6%; p-value=0.85), DistilBERT (1.5%; p-value=0.49), and RoBERTa (8.7%; p-value=0.51); and PRISM dataset - ALBERT (6.2%; p-value=0.37), BART (6%; p-value=0.01), BERT (2.3%; p-value=0.02), BioBERT (5%; p-value=0.007), DistilBERT (6.2%; p-value=0.007), and RoBERTa (6.2%; p-value=0.007).

bioRxiv preprint doi: https://doi.org/10.1101/2025.02.27.640661; this version posted March 3, 2025. The copyright holder for this preprint (which was not certified by peer review) is the author/funder, who has granted bioRxiv a license to display the preprint in perpetuity. It is made available under aCC-BY 4.0 International license.

Supplementary Note 10: Few-Shot Prompting based on Structure Similarity of Drugs

Drugs with similar chemical structure have shown to exhibit similar sensitivity patterns (Zhang et al. 2015). We carried out a subanalysis wherein we probe if exploiting this phenomenon in GPT-3's in-context learning would refine its predictive power. We first computed the Tanimoto coefficients between the drugs' SMILES strings to find the structurally similar drug compounds in our dataset, as visualized in **Figure 6B(i)** for 15 drugs from the DrugComb dataset. We then test GPT-3 for 10-shot inference on each drug by providing five 'sensitive' in-context examples in the form of drug's name, cell line's name and drug's SMILE, that are associated with the corresponding structurally similar drugs (high Tanimoto score). While five 'resistant' examples are also provided but from the not similar drugs (low Tanimoto score). The results are reported in **Figure 6B(ii)** for 13 of the 15 drugs and compared against performance with randomly selected in-context examples (Random); drugs 5-fluorouracil and vorinostat were excluded from the evaluation as they have low Tanimoto scores with the other drugs. The results suggest that prompting GPT-3 with the context of structural similarity acts as inductive bias that could improve its generalization capability, as majority of the drugs (9 out of 13) had boosted performances by including carefully selected demonstration examples - filtered through Tanimoto coefficient rather than randomly sampled - although the differences are not statistically significant (p-value=0.33).

bioRxiv preprint doi: https://doi.org/10.1101/2025.02.27.640661; this version posted March 3, 2025. The copyright holder for this preprint (which was not certified by peer review) is the author/funder, who has granted bioRxiv a license to display the preprint in perpetuity. It is made available under aCC-BY 4.0 International license.

Supplementary Table 1: Summary of benchmark pharmacogenomics datasets across tissue types using different feature combinations. We used the following abbreviations: BI (basic information), MS (molecular structure information), MGC (additional molecular or genomic context).

	GDSC	CCLE	DrugComb	PRISM
**# Drug-Cell Line Input Pairs (BI)**				
Lung	15653	1220	136690	69927
Thyroid	4037	88	157602	7424
Breast	13106	373	114757	14986
Brain	3617	402	84921	24363
Colon/Stomach	12538	447	2414	18807
**BI + MS**				
Lung	9761	579	31794	69927
Thyroid	2509	43	15989	7424
Breast	8059	188	31359	14986
Brain	2632	185	12696	24363
Colon/Stomach	7367	222	913	18807
**BI + MGC**				
Lung	14816	883	31794	69927
Thyroid	529	68	15989	7424
Breast	12540	323	31359	14986
Brain	2979	278	12696	24363
Colon/Stomach	12110	256	913	18807
**BI + MS + MGC**				
Lung	9278	412	31794	69927
Thyroid	448	32	15989	7424
Breast	7728	164	31359	14986
Brain	2148	127	12696	24363
Colon/Stomach	7133	127	913	18807

bioRxiv preprint doi: https://doi.org/10.1101/2025.02.27.640661; this version posted March 3, 2025. The copyright holder for this preprint (which was not certified by peer review) is the author/funder, who has granted bioRxiv a license to display the preprint in perpetuity. It is made available under aCC-BY 4.0 International license.

Supplementary Table 2: Class distributions in the training sets of datasets across tissues.

		Sensitive	*Resistant*
**GDSC**	**Lung**	1414	11108
	**Thyroid**	372	2857
	**Breast**	1236	9248
	**Brain**	357	2536
	**Colon/Stomach**	1237	8793
**CCLE**		*Sensitive*	*Resistant*
	**Lung**	177	799
	**Thyroid**	16	54
	**Breast**	53	245
	**Brain**	59	262
	**Colon/Stomach**	65	292
**DrugComb**		*Sensitive*	*Resistant*
	**Lung**	36502	72850
	**Thyroid**	87928	38153
	**Breast**	31243	60562
	**Brain**	24152	43784
	**Colon/Stomach**	315	1616
**PRISM**		*Sensitive*	*Resistant*
	**Lung**	8438	47503
	**Thyroid**	988	4951
	**Breast**	1689	10299
	**Brain**	2890	16600
	**Colon/Stomach**	2321	12724

bioRxiv preprint doi: https://doi.org/10.1101/2025.02.27.640661; this version posted March 3, 2025. The copyright holder for this preprint (which was not certified by peer review) is the author/funder, who has granted bioRxiv a license to display the preprint in perpetuity. It is made available under aCC-BY 4.0 International license.

Supplementary Table 3: Top three Afatinib treated sensitive and resistant cell lines from the GDSC dataset.

	Cell Line	Tissue
Sensitive	MDAMB175VII	Breast
	UACC893	Breast
	NCIH508	Stomach
Resistant	HTCC3	Thyroid
	NCIH1651	Lung
	SNUC1	Stomach

bioRxiv preprint doi: https://doi.org/10.1101/2025.02.27.640661; this version posted March 3, 2025. The copyright holder for this preprint (which was not certified by peer review) is the author/funder, who has granted bioRxiv a license to display the preprint in perpetuity. It is made available under aCC-BY 4.0 International license.

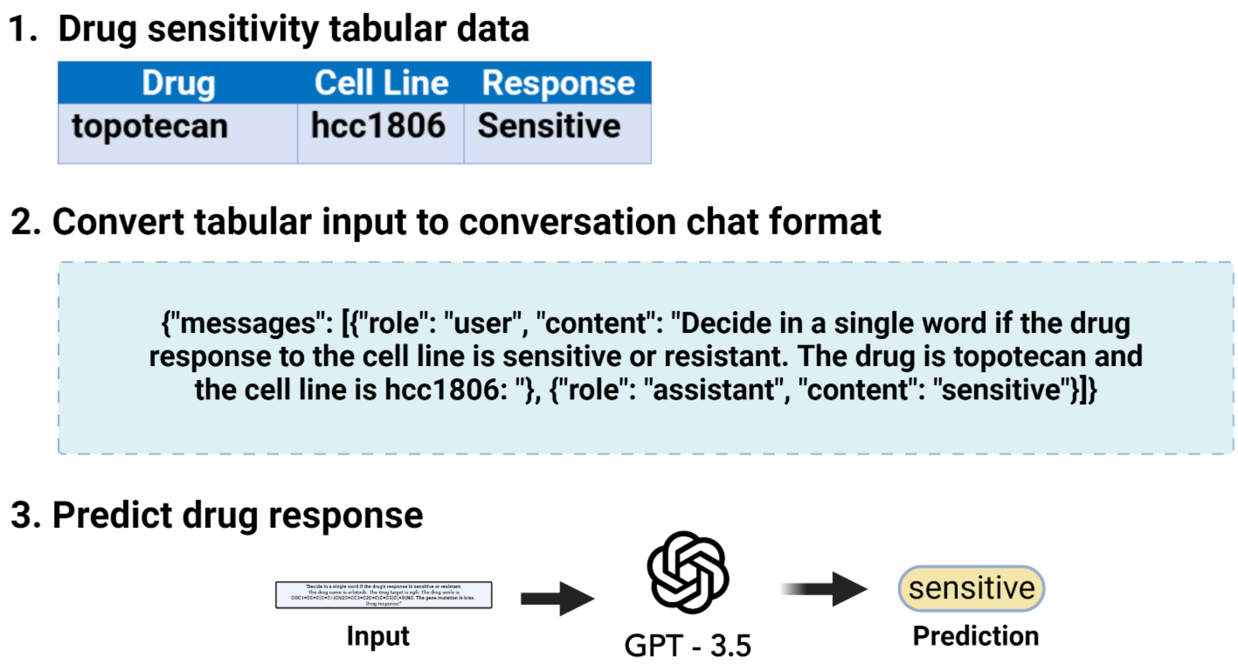

Supplementary Figure 1: Example demonstrating the conversation chat format for preparing the training samples for fine-tuning with GPT-3.5 and GPT-4.

bioRxiv preprint doi: https://doi.org/10.1101/2025.02.27.640661; this version posted March 3, 2025. The copyright holder for this preprint (which was not certified by peer review) is the author/funder, who has granted bioRxiv a license to display the preprint in perpetuity. It is made available under aCC-BY 4.0 International license.

(A) Instruction Prompt

"Decide in a single word if the drug response is sensitive or resistant.
The drug name is daporinad. The cell line is vmrc-lcd. Drug response: sensitive
The drug name is uprosertib. The cell line is nci-h1693. Drug response: resistant
The drug name is bortezomib. The cell line is lc-2-ad. Drug response:"

(B) Instruction-Prefix Prompt

"Decide in a single word if the drug response is sensitive or resistant.
Drug name: daporinad; cell line: vmrc-lcd; drug response: sensitive
Drug name: uprosertib; cell line: nci-h1693; drug response: resistant
Drug name: bortezomib; cell line: lc-2-ad; drug response:"

(C) Cloze Prompt

"The cell line vmrc-lcd is sensitive to the drug daporinad.
The cell line nci-h1693 is resistant to the drug uprosertib.
The cell line lc-2-ad is [Z] to the drug bortezomib."

Supplementary Figure 2: Examples illustrating different prompt templates as input for few-shot evaluation.

bioRxiv preprint doi: https://doi.org/10.1101/2025.02.27.640661; this version posted March 3, 2025. The copyright holder for this preprint (which was not certified by peer review) is the author/funder, who has granted bioRxiv a license to display the preprint in perpetuity. It is made available under aCC-BY 4.0 International license.

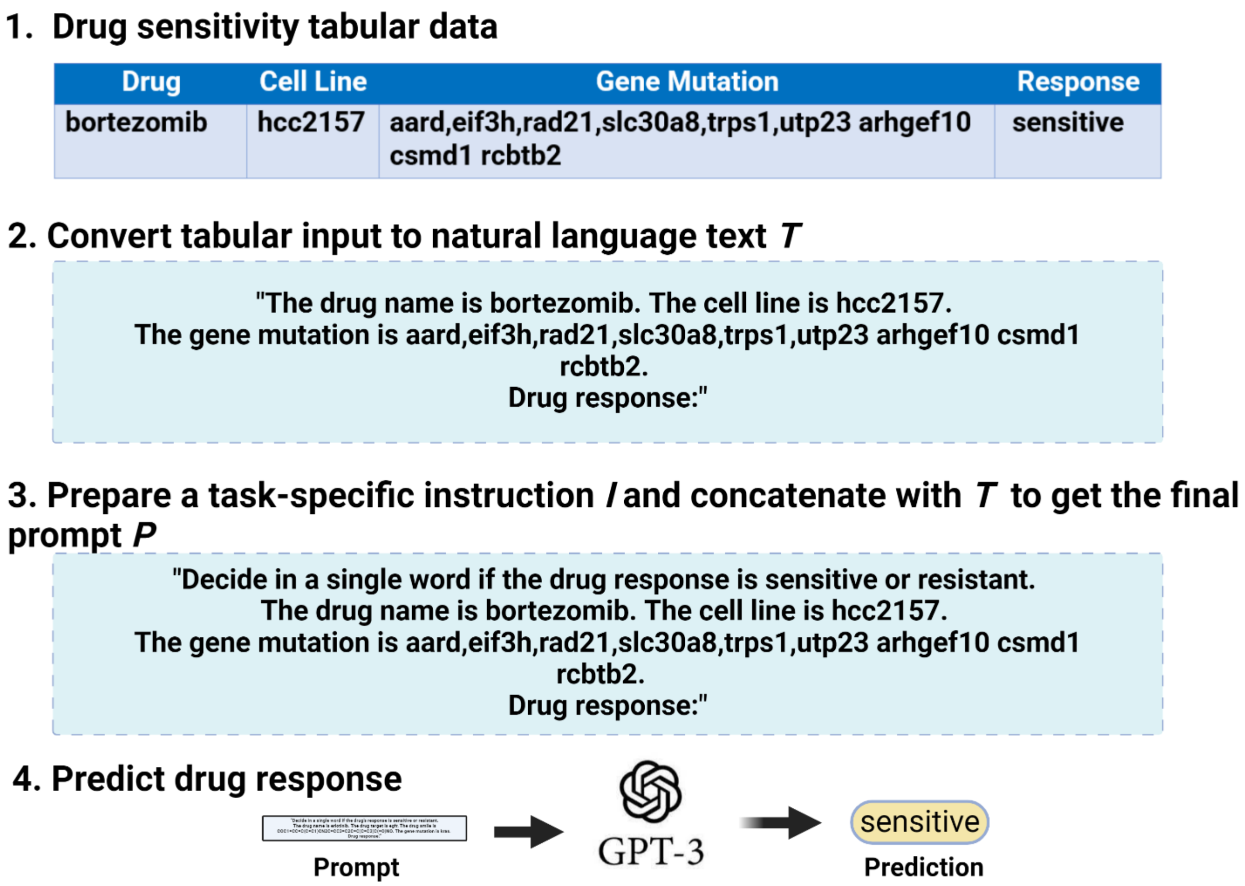

Supplementary Figure 3A: Input prompt example containing feature MGC (BI + MGC) from GDSC dataset.

bioRxiv preprint doi: https://doi.org/10.1101/2025.02.27.640661; this version posted March 3, 2025. The copyright holder for this preprint (which was not certified by peer review) is the author/funder, who has granted bioRxiv a license to display the preprint in perpetuity. It is made available under aCC-BY 4.0 International license.

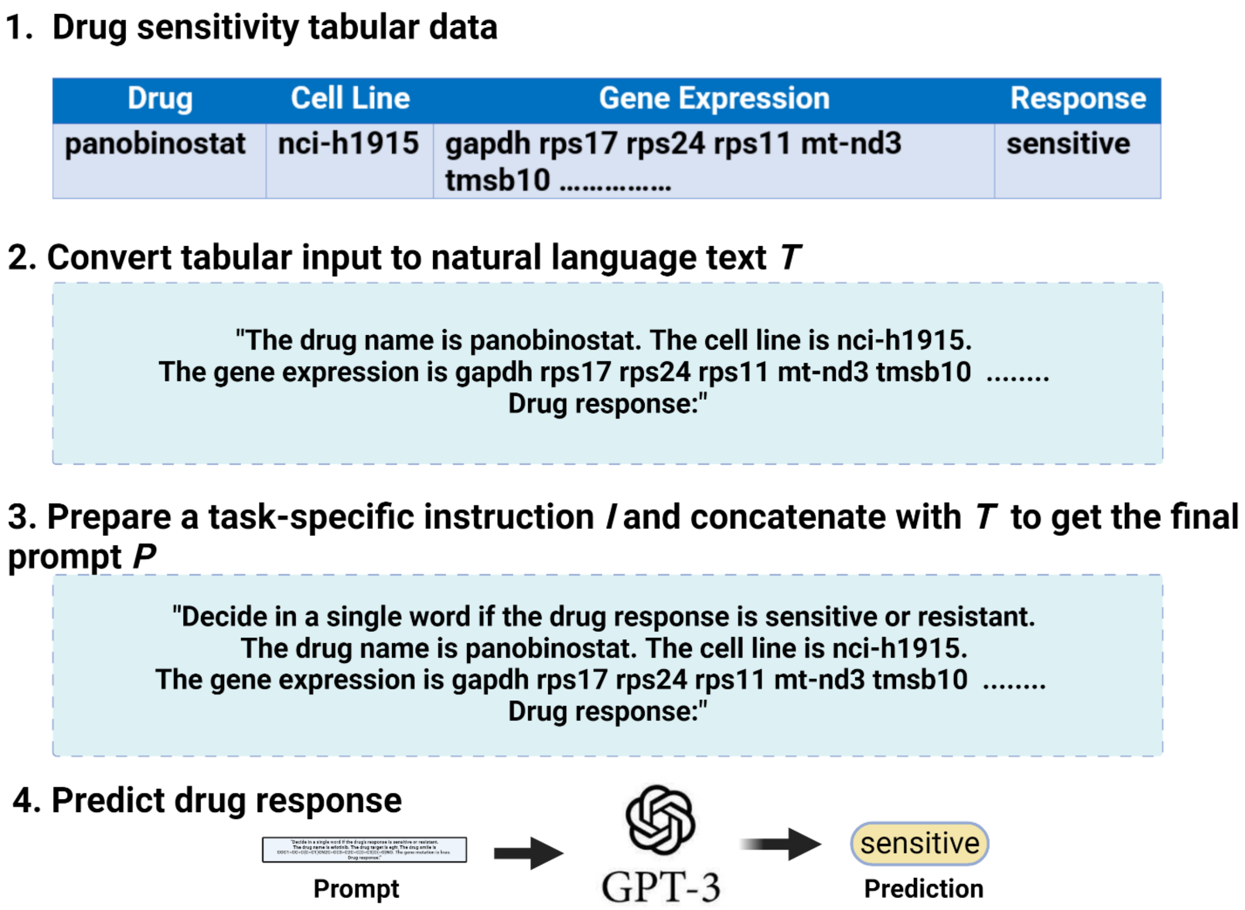

Supplementary Figure 3B: Input prompt example containing feature MGC (BI + MGC) from CCLE dataset. Part of the gene expression is replaced here with ellipsis for brevity.

bioRxiv preprint doi: https://doi.org/10.1101/2025.02.27.640661; this version posted March 3, 2025. The copyright holder for this preprint (which was not certified by peer review) is the author/funder, who has granted bioRxiv a license to display the preprint in perpetuity. It is made available under aCC-BY 4.0 International license.

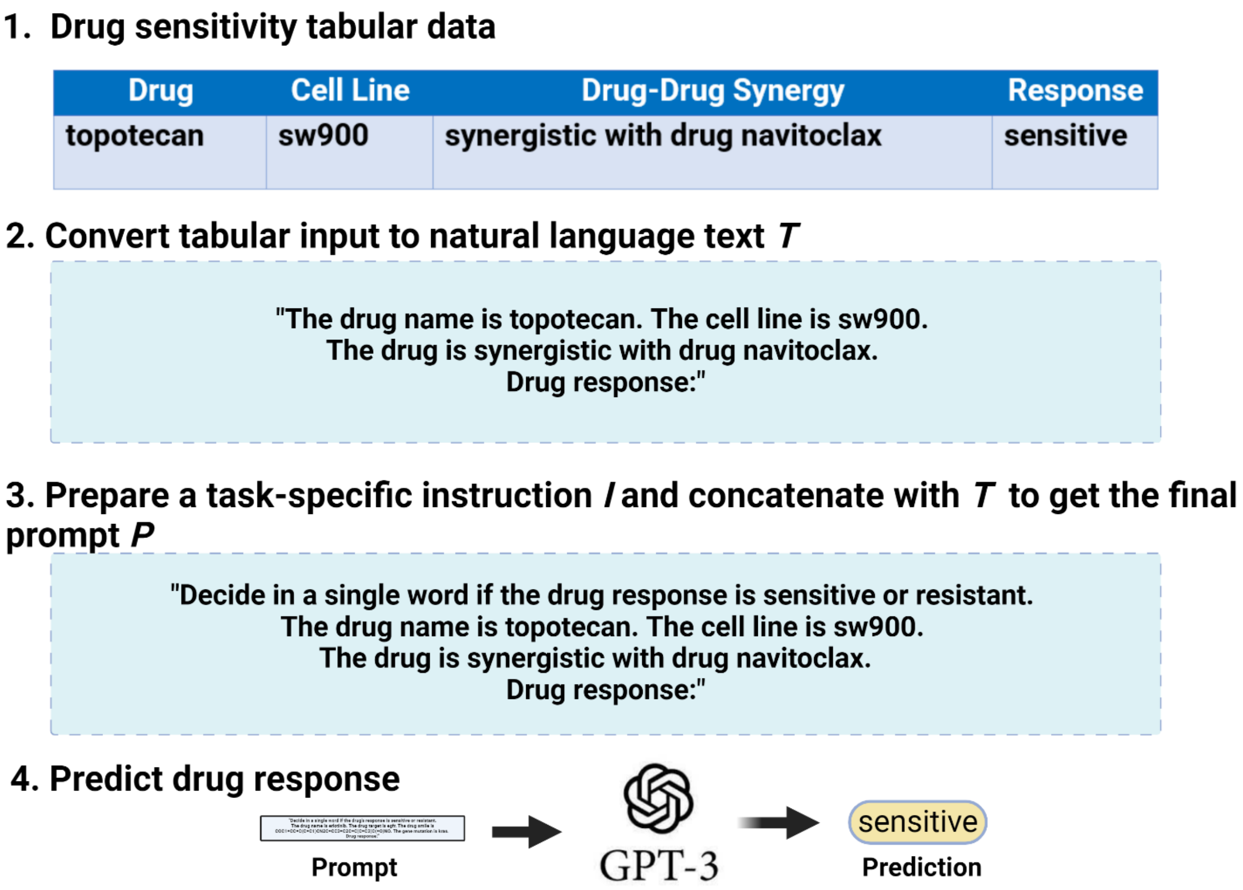

Supplementary Figure 3C: Input prompt example containing feature MGC (BI + MGC) from DrugComb dataset.

bioRxiv preprint doi: https://doi.org/10.1101/2025.02.27.640661; this version posted March 3, 2025. The copyright holder for this preprint (which was not certified by peer review) is the author/funder, who has granted bioRxiv a license to display the preprint in perpetuity. It is made available under aCC-BY 4.0 International license.

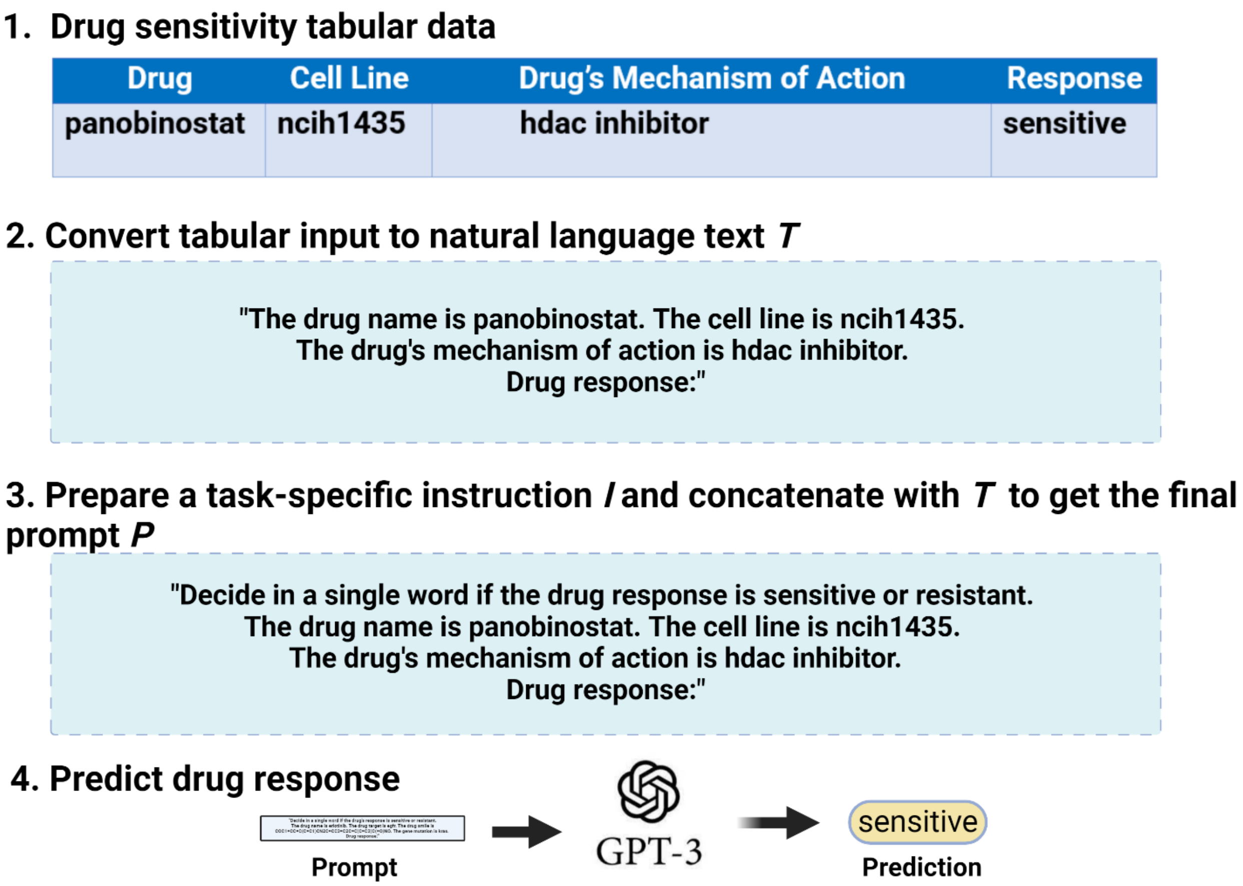

Supplementary Figure 3D: Input prompt example containing feature MGC (Drug + BI + MGC) from PRISM dataset.

bioRxiv preprint doi: https://doi.org/10.1101/2025.02.27.640661; this version posted March 3, 2025. The copyright holder for this preprint (which was not certified by peer review) is the author/funder, who has granted bioRxiv a license to display the preprint in perpetuity. It is made available under aCC-BY 4.0 International license.

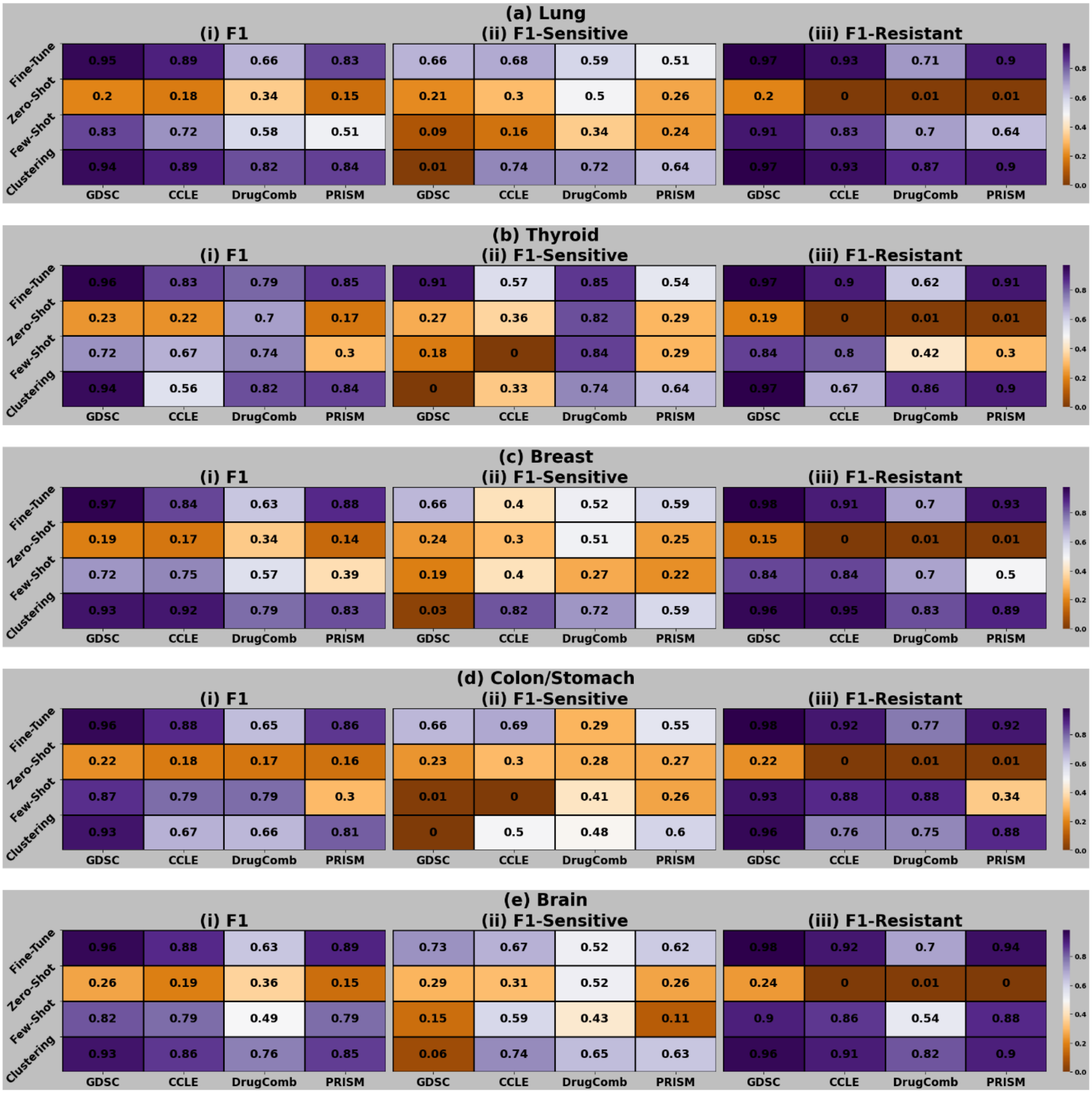

Supplementary Figure 4: Performance comparisons among learning paradigms across datasets per tissue type.

bioRxiv preprint doi: https://doi.org/10.1101/2025.02.27.640661; this version posted March 3, 2025. The copyright holder for this preprint (which was not certified by peer review) is the author/funder, who has granted bioRxiv a license to display the preprint in perpetuity. It is made available under aCC-BY 4.0 International license.

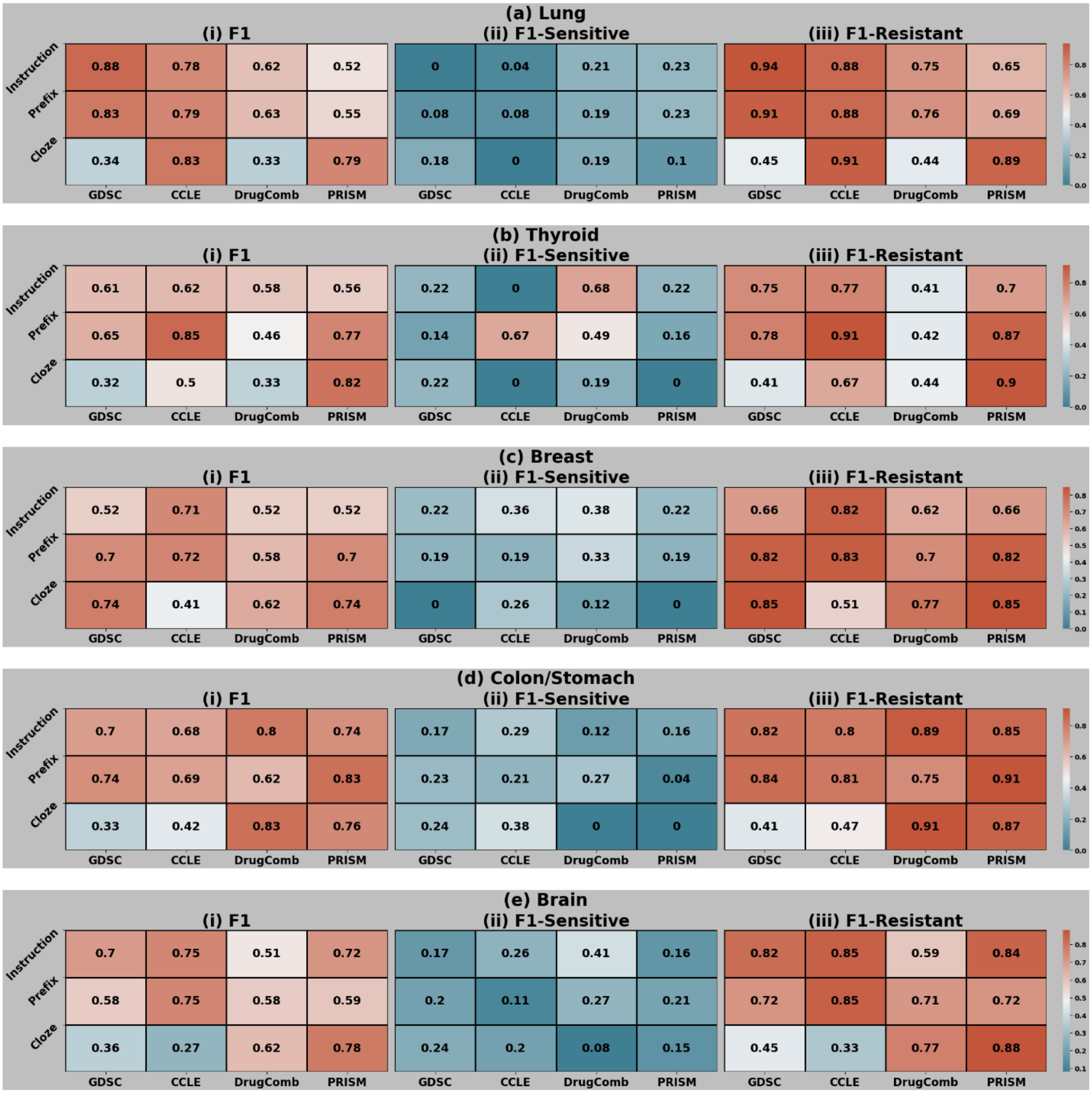

Supplementary Figure 5: Performance comparisons of prompt templates across datasets per tissue type.

bioRxiv preprint doi: https://doi.org/10.1101/2025.02.27.640661; this version posted March 3, 2025. The copyright holder for this preprint (which was not certified by peer review) is the author/funder, who has granted bioRxiv a license to display the preprint in perpetuity. It is made available under aCC-BY 4.0 International license.

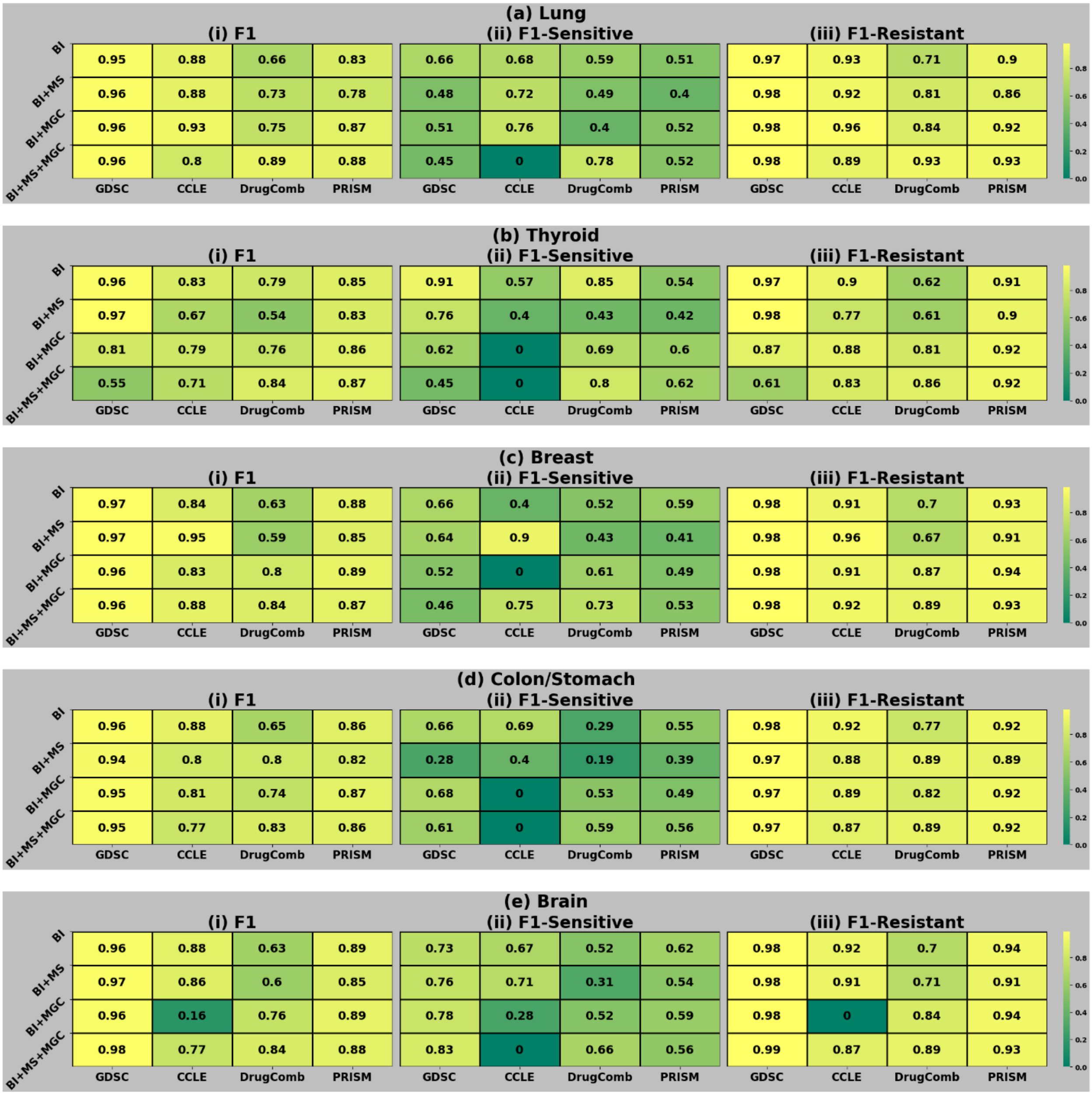

Supplementary Figure 6: Performance evaluation on feature combinations per tissue type across datasets.

bioRxiv preprint doi: https://doi.org/10.1101/2025.02.27.640661; this version posted March 3, 2025. The copyright holder for this preprint (which was not certified by peer review) is the author/funder, who has granted bioRxiv a license to display the preprint in perpetuity. It is made available under aCC-BY 4.0 International license.

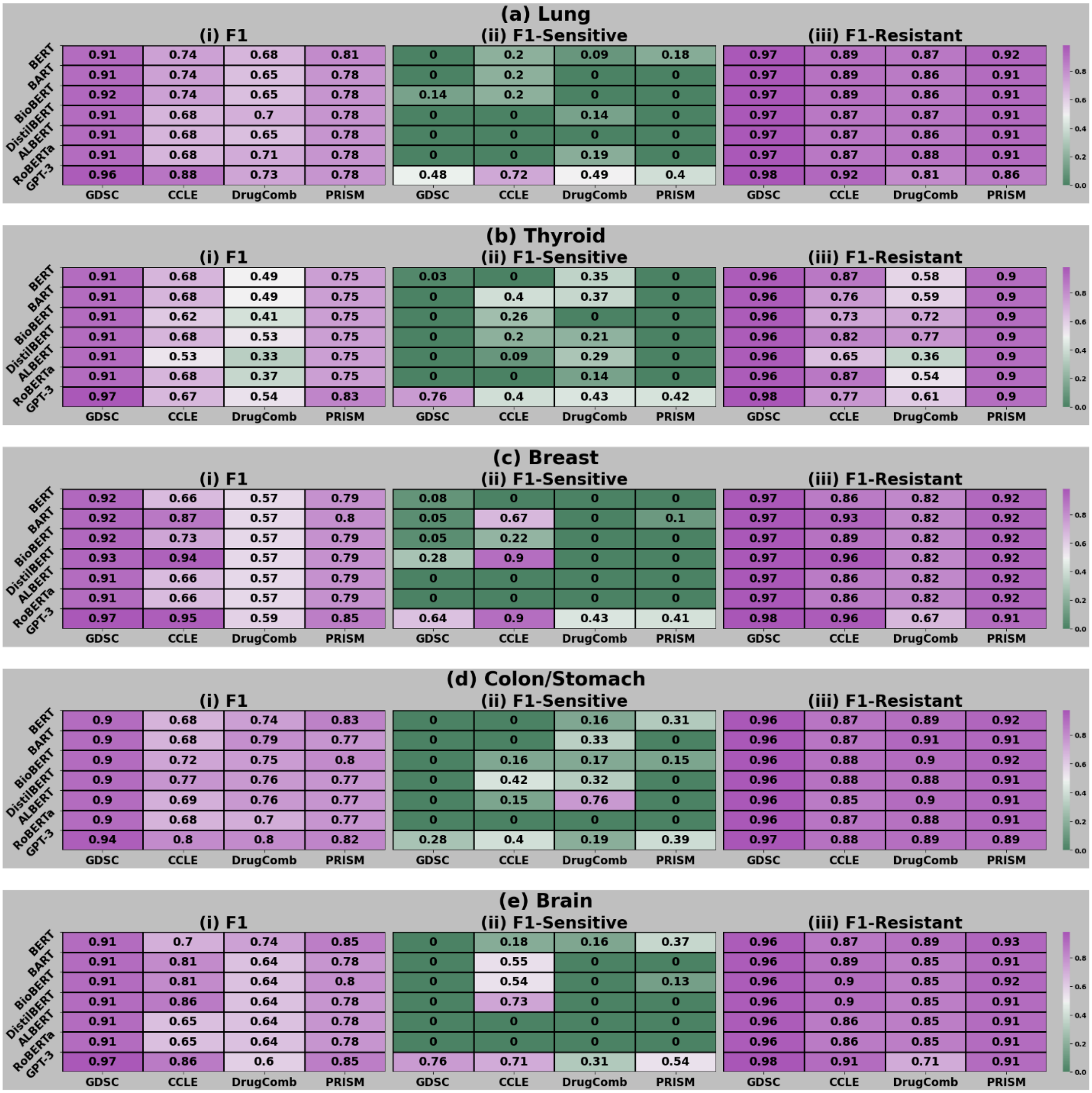

Supplementary Figure 7: Baseline performance comparisons of GPT-3 with other language models.

bioRxiv preprint doi: https://doi.org/10.1101/2025.02.27.640661; this version posted March 3, 2025. The copyright holder for this preprint (which was not certified by peer review) is the author/funder, who has granted bioRxiv a license to display the preprint in perpetuity. It is made available under aCC-BY 4.0 International license.

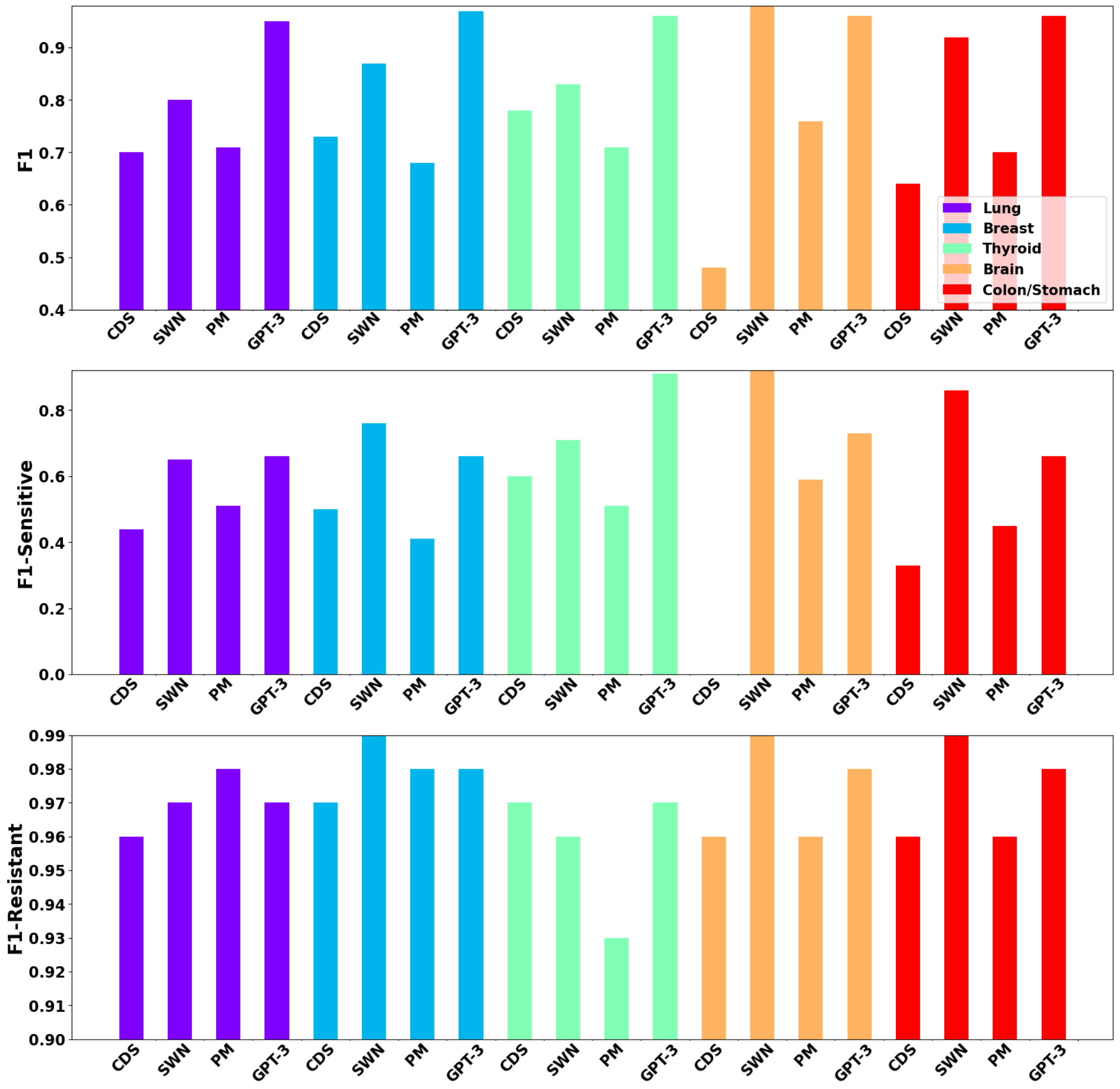

Supplementary Figure 8: Baseline performance comparisons of GPT-3 with other drug response models.
